# Drug–herb interactions: a challenge and clinical concern in primary healthcare

**DOI:** 10.3389/fmed.2025.1657005

**Published:** 2025-11-07

**Authors:** Sujithra M., Rammanohar Puthiyedath, Zeena S. Pillai

**Affiliations:** 1 Amrita School of Ayurveda, Amrita Vishwa Vidyapeetham, Kollam, India; 2 Department of Chemistry, Amrita Vishwa Vidyapeetham, Kollam, India

**Keywords:** drug–herb interactions, primary healthcare, community health, family medicine, patient safety

## Abstract

Primary healthcare (PHC) is the first level of care that provides basic medical services to people in their own communities. Family doctors, local clinics, and community health centers fall under this category. When it comes to drug–herb interactions, PHC faces unique challenges. Herbal medicine usage is increasing globally, raising the challenge of drug–herb interactions due to simultaneous administration with modern pharmacological agents. Literature on drug–herb interactions is growing but inadequately explored in the context of primary healthcare. This mini review focuses on the concerns and challenges of drug–herb interactions in a primary healthcare setup, taking into consideration patterns, high-risk scenarios, prediction and assessment, and management strategies. It highlights the obstacles faced by primary healthcare practitioners, including time and resource limitations, resource and knowledge gaps, and difficulties in communication. In addition, it emphasizes the need for organized approaches to screen the interactions using risk assessment tools created for primary care and to enhance educational resources to ensure patient safety and medical outcomes in a community-based healthcare setting. A consolidated evidence matrix of drug–herb interactions from published research articles is included, which can serve as a one-stop reference resource at the point of care.

## Introduction

Primary healthcare is the foundation of healthcare delivery, offering easily accessible, comprehensive, and orchestrated care to varied patient populations. It focuses on intersectoral equity, participation, solidarity, and social justice collaboration (1–4). It also encompasses real-life struggles anxieties, chronic diseases, mortality, and healthcare decisions people face. Countries with advanced PHC generate the best health outcomes. Across the world, people are drawn to traditional medicines to tackle their health conditions. For centuries, diverse traditional systems of medicine (Ayurveda, Chinese, Siddha, Iranian, Unani, Acupuncture, Korean, Ifa, and African) have used herbs as primary ingredients in medications. These herbs are storehouses of phytochemicals, also known as secondary metabolites. Phytochemicals have the potential to interact with modern pharmacological agents, sometimes leading to adverse effects. It is estimated that approximately 80% of people worldwide use herbal medicine (5), depending on cultural context and geographic location. This widespread use adds complexity to the PHC ecosystem, necessitating systematic approaches and carefully thought-out initiatives to ensure patient safety. Some countries, such as the Republic of Korea, have already integrated traditional medicine with conventional medicine within their PHC systems (6–8).

A cross-sectional survey conducted on 400 adults in England, along with a patient cohort database at the American University of Beirut Medical Center of the ambulatory clinics (9, 10), indicated that the patient population visiting PHC facilities often suffers from multiple comorbidities, has simultaneous consultations with physicians of different medical systems, and practices polypharmacy. This sets the stage for drug–herb interactions that can go undetected and significantly impact the treatment outcomes and patient safety. In this scenario, it is imperative for healthcare providers at the PHC level to take measures to screen patients for the use of herbal medications and the potential drug–herb interactions. However, the majority of PHC providers do not have adequate knowledge of herbal medicine or its pharmacology (11).

The consequences of undetected interactions extend way beyond the harm to the individual patient to a broader arena, implying treatment failures, emergency department visits, and healthcare expenditure. Drug–herb interactions (DHIs) can cause toxicity, adverse effects, or even undesired therapeutic outcomes (12). This review synthesizes published, peer-reviewed studies that are clustered into thematic sections for better understanding. It is unique in adopting a primary healthcare (PHC) lens focusing on the operational realities of PHC settings rather than taking a general approach. Supplementary data include a summary of updated research on DHIs from published literature covering prominent herbs and biomedicine.

## Prevalence and patterns in primary care

### Patient demography and usage patterns

Traditional practitioners and herbal medications remain much sought after as the tools for relief from and cure of several diseases across the world. The use of traditional medical systems has been approved by the WHO for inclusion into PHC (13). The most vulnerable group in this setting is the elderly patients, as they are on multiple medications for chronic conditions and simultaneously consume herbal medicines, which have been a part of their healthcare routine for decades. This population combines herbal medications with prescription medicines, not realizing the possibility of drug–herb interactions, thus compromising treatment benefits or increasing the risk of adverse events (14–16). Some examples of herbal supplements used for specific health benefits include bitter melon or cinnamon for managing diabetes, garlic for improving cardiovascular health, and various adaptogenic herbs for maintaining general health and vitality (17–19).

Expatriate populations further increase the complexity of the PHC setting, as prescription medicines are often consumed alongside traditional medicines. Such concurrent medication practices add to the concerns about adverse drug reactions (20). Published research demonstrates that medical students also consume herbal medicines with little or no knowledge about such medicines and the potential side effects (21). In several countries, such as Slovenia, there is an extensive tradition of using herbal medicines (22).

### Primary healthcare and common herbal products

Herbal products are plants and plant-derived substances (23). Herbs are the main ingredients in several traditional medical formulations in different practices of healing (24). The therapeutic efficacy of herbal medicines has been supported by research in various health conditions such as respiratory ailments, pain and inflammation, digestive complaints, and immune dysregulation (25). The phytochemicals vary from herb to herb, making it difficult to easily predict potential drug–herb interactions. For example, *Echinacea* is used to boost the immune system, ginger for digestive issues, and *Ginkgo biloba* for cognitive function (26). Phytoecdysteroid-containing herbal products (derived from spinach and quinoa) are used by sportsmen and bodybuilders (27). Garlic is one of the most sought-after food supplements to manage various health conditions, including cardiovascular diseases. The antiplatelet, pro-circulatory, and hypolipidemic effects of garlic are well-studied and researched. It is also a known hepatoprotective and anti-cancerous agent with a probable immunomodulating activity. Available forms of garlic range from raw cloves to extracts of aged garlic, garlic oil, and oil macerate (28). The other commonly used herb in primary healthcare is St. John’s wort as a popular antidepressant (29). A total of 507 ginseng-containing commercial herbal products are being sold across 6 continents in 12 countries (30). Ginseng is reported to have interactions with insulin, anticoagulants, digoxin, and monoamine oxidase inhibitors (31). Turmeric is consumed for its potential health benefits in heart disease, arthritis, Alzheimer’s disease, gastrointestinal disorders, and metabolic syndrome, with evidence obtained from preclinical studies (32). Ephedra alkaloids are combined with caffeine and used to enhance metabolism. Although ephedra extracts are used in small quantities, 64% of herb-related adverse reactions related to ephedra were reported to the California Poison Control Center (33).

### Disclosure patterns

Patient disclosure patterns regarding herbal medicine use represent a critical challenge in the PHC settings, with significant implications for identifying and managing potential drug–herb interactions. A national survey conducted in the United States identified that 19% of adults used herbal products and dietary supplements, and this could be even higher in the ethnic communities (34). Data suggest that only 23–37% of Complementary and Alternative Medicine (CAM) users disclosed at least one type of CAM to their physician (35, 36). The herbal-drug/supplement (HDS) disclosure rates are influenced by ethnic variations, with Asians and Asian Americans having a possibility of inadequate disclosure. Studies indicate that Hispanic and Asian Americans exhibit a low rate of disclosure of HDS (21 to 31%) (37, 38). There is also variation in the disclosure pattern depending on the age, number of visits, gender, education, income, geographical region, insurance, self-rated health, use of prescription medications, and the source of the medicine (39). This non-disclosure phenomenon could be attributed to both patient and physician characteristics (40–42). Poor communication is a major problem in PHC, to the extent that a shared decision is very difficult. As PHC is a user-centered healthcare model, complete disclosure is beneficial (43).

A survey of 59 attending physicians, 57 resident physicians, and 26 medical students concluded that they had minimal knowledge about interactions and toxicities. Healthcare providers have limited training in DHIs, and this supports the fact that they may not even ask the patients for the details of HDS consumption (44). Healthcare providers often lack sufficient training in herbal medicine pharmacology and may not routinely inquire about supplement use during patient consultations. Disclosure has been increasingly identified as a central challenge facing patient management to prevent DHIs (45). The potential risks go undetected at the earliest or most critical intervention point. If disclosure rates can be increased, the associated potential risks of interaction can be brought down significantly (46).

### Patient communication barriers

Inadequate communication with the patients will have consequences with regard to patient safety and survival. This observation is supported by the risk management literature, where 70% of adverse events are connected to communication errors (47). A conversation should be initiated with the patients to get the complete medical history. Patients should be counseled about the usage of herbs with an empathetic approach by the clinician. Efforts should be made to enhance patient awareness concerning interactions or adverse reactions and the appropriate use of the herbal supplements (48).

### High-risk clinical scenarios in primary care

In primary care, management of chronic diseases presents a significant challenge, concerned with drug–herb interactions. For centuries, plants were an important source of antidiabetic drugs (49). Patients on insulin or sulfonylureas who consume chromium supplements, bitter melon, and fenugreek will drop to dangerously low levels of blood sugar, owing to the enhanced hypoglycemic effects (50). Ginseng affects the blood sugar, but variation was observed across batches, varieties, species, and preparation (51). This makes the hypoglycemic effect due to DHIs unpredictable.

Another clinical condition that is a matter of concern is cardiovascular disease. Several herbs, such as garlic (52), ginkgo (53), or dong quai supplements (54), are concurrently consumed by patients on blood thinners and anticoagulants such as warfarin. As the anti-coagulation effect increases, the risk of bleeding increases. Licorice potentiates the oral and topical corticosteroids when taken concurrently (52). This can lead to a health crisis. When a single dose of hawthorn was administered along with digoxin, there was no significant difference in the pharmacokinetics of digoxin. However, there is no information on the outcome when the dose of hawthorn is increased (55). In this case, the information is incomplete.

The best example is the interaction between selective serotonin reuptake inhibitors (SSRIs) and St. John’s wort (56), which can cause serotonin syndrome, which is a potentially life-threatening clinical condition. The symptoms include autonomic instability, altered mental status, and neuromuscular anomalies. The concurrent consumption of benzodiazepine and green tea alters the benzodiazepine pharmacokinetics (57). When cannabis is simultaneously consumed with non-steroidal anti-inflammatory drugs (NSAIDs) and SSRIs, extreme caution should be taken, as it modulates the antidepressant effect, similar to citalopram (58). As the physician has no time to screen the use of supplements, this can have dire consequences. The risk of interactions is specifically high in primary care, as the practice of polypharmacy is on the rise. In patients on multiple medications, several CYP450 substrates may compete with the enzyme inhibitors or inducers of herbs, leading to unpredictable variations in drug metabolism. When the pathways of the medicines and herbal supplements are identical, it can cause an additive effect that can lead to undesirable consequences. Polypharmacy is closely connected to adverse events including drug reactions, mortality, falls, increased hospitalization, and recurrence of symptoms (59–61).

### Detection and assessment challenges

In a PHC setting, there are no routine questions asked that would help identify possible interactions during medication reconciliation (62). If at all, DHI has been detected, it may or may not be reported at all. In the majority of the cases, the observed phenomenon cannot be explained plausibly, and there is uncertainty about causality (63).

Establishing the identity of the herb and identifying the phytochemicals responsible for DHI adds to the challenge. The misidentification of the herbs is attributed to morphological similarities, references, and local usage. The challenge is even more convoluted, owing to variation in nomenclature due to linguistic diversity (64). The phytochemicals, such as sterols, alkaloids, fatty acids, glycosides, flavonoids, tannins, saponins, phenolics, and terpenes, are very potent therapeutic agents (63). There are hundreds of organic chemicals in the extract of a single plant. For example, from *Panax* *ginseng*, more than 28 ginsenosides were extracted, and each of them is associated with different therapeutic effects. Several herbs are currently being researched to understand and unravel the mechanism of action (65, 66). Accurate detection of DHI is a challenge when the pathway of action of the phytochemicals is unknown.

**Figure 1:**
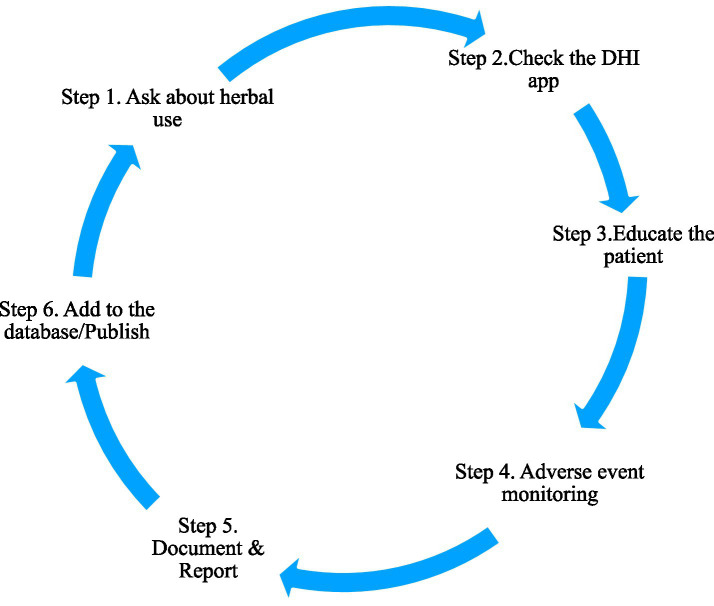
Simple protocol to be followed in a PHC setting to address DHI.

### Operational constraints

In primary care, the existing environment is completely different from what is available in specialty clinics, creating challenges to address drug–herb interactions. There is a communication gap in history taking and dissemination of knowledge regarding the regular medications taken by the patient, owing primarily to the lack of time (67). The building pressure of time constraints burns the physician out, affecting the metrics and outcomes (68). The primary target is disease management while balancing the assessment of drug–herb interaction, regular monitoring of chronic diseases, prophylactic approaches, mental health screening, and activities for health maintenance. When a drug–herb interaction occurs, the adverse events that unfold are also to be managed by the healthcare provider. The risks must be identified and assessed, strategies must be developed and adopted to reduce the risks, and evaluation must be made a top priority (69).

### Existing knowledge and gaps in training

The majority of the physicians trained in the West are not aware of the risks and benefits of the primary healthcare setup (70). A physician can become a reliable and informed healthcare provider only by being well educated about CAM, especially owing to the increased popularity of CAM (71). Physicians have limited knowledge about CAM use, owing to insufficient exposure (72). It has been observed that physicians underestimate the use of complementary medicine by their patients (73).

### Information resource challenge

Herbs are referred to as “natural,” and the general belief is that they cannot cause any harm, leading to their widespread use without caution (74). The commercialization of the herbal products is happening so rapidly that doctors and pharmacists find it difficult to validate their use. The problem is further aggravated by knowledge gaps about regulation and labeling (75). Knowledge-based approaches can be followed to address the extensive amount of information available on DHIs. For example, Kinney (76) developed an expert system that used a microcomputer to assess interactions in patients who were hospitalized. In addition, taking into consideration the risks involved, studies state that an absence or decreased level of knowledge of interactions can lead to deleterious and fatal outcomes (77).

### Evidence quality levels

There are significant challenges faced by PHCs in evidence quality for drug–herb interactions owing to the research limitations, gaps in methodology, and barriers in conducting a systematic investigation. The researchers must emphasize the need for population-based, large-scale studies to create standardized assessment protocols and models that include traditional systems and the public to completely understand the pattern of usage. By doing so, appropriate patient awareness programs considering cultural practices can be developed. Figure 1 suggests a simple protocol to be followed in the PHC settings to address DHI.

### Recent advancements in drug–herb interaction management

The recent developments in drug–herb interaction management depict both technological advancement and the challenges in the protocol implementation. Specialized medication reconciliation tools are currently a part of the enhanced electronic health record (EHR) systems. There are separate apps for herbal supplements to check the possibility of interactions, supported by the Agency for Healthcare Research and Quality (AHRQ)-funded digital health initiatives. Medication reconciliation requirements have been reinforced, thus creating several safety check modules. The disclosure rates and patterns of usage have been updated, and the current data reveal that they are very low at 25–33% (78).

There is also disparity among the Asian and Asian American populations (21–31%). Numerous initiatives in policy such as the draft guidance on labeling of drug interactions by the FDA’s (79) ICH M12 have also emerged. The pharmacovigilance systems enhanced by systematic reviews analyzing adverse events were published in August 2024 (80). Database performance was improved using UpToDate Lexidrug, obtaining 0.98 positive predictive values for the identification of DHIs. In spite of all these efforts, there still exist gaps, specifically in a primary healthcare setting, that need to be addressed.

### Scope and boundaries of the review

This mini review focuses only on PHC settings and the multifaceted challenges owing to DHIs in a community clinical environment. The investigation encompasses major domains: patterns of usage and behavior, diagnostic complexities, and barriers and constraints across various healthcare systems. To have a consistent methodological rigor, the review excludes unpublished institutional reports, individual anecdotes, and non-peer-reviewed literature. The supplementary evidence data were compiled systematically exclusively through searches in the PubMed database. The commonly used herbs with documentation on concurrent use with modern drugs in clinical practice were included.

### Future directions and recommendations

Population-based studies in PHC settings are the need of the hour to authenticate screening protocols and enhance EHR documentation systems. Herbal pharmacology training must become mandatory in medical education curricula, not an optional supplement. At every PHC center, validated patient questionnaires on supplement use should be incorporated to create minimum competency standards and DHI-specific quality metrics. The evidence is irrefutable: structured change from inconsistent provider-dependent assessment to standardized protocols is crucial for patient safety. The drug–herb interaction matrix in Supplementary Table 1 is an example of how a ready reckoner can be created and regularly updated to aid PHC physicians to proactively identify DHIs. Immediate access to data is possible without literature searches.

## Conclusion

DHIs present as a growing concern, owing to multiple complexities. Data from clinical trials, both *in vivo* and *in vitro*, should be considered for use with caution. Patient and physician education and awareness regarding DHIs in the PHC setting are mandatory to avoid fatal outcomes.

## Supplementary material

SUPPLEMENTARY TABLE 1 Updated published research on drug-herb interaction, from PubMed (added as supplement).
