# Supplementary material for: Drug–herb interactions: a challenge and clinical concern in primary healthcare
Source: Front Med (Lausanne). 2025 Nov 7;12:1657005. doi: 10.3389/fmed.2025.1657005 (PMC12634587; doi:10.3389/fmed.2025.1657005)
Supplement: SUPPLEMENTARY TABLE 1 — Updated published research on drug-herb interaction, from PubMed (added as supplement). [file Table_1.docx]

| **1`S No** | **Plant source** | **Type of Article** | **Ayurvedic nomeclature** | **Interaction with** | **Effect** | **Reference Articles** |
| --- | --- | --- | --- | --- | --- | --- |
| 1 | *Allium sativum* L. ( Garlic) | Review | Laśūna | Warfarin | Bleeding | Fugh-Berman A. Herb-drug interactions. Lancet. 2000 Jan 8;355(9198):134-8. |
|  |  | Review |  |  | Increased Clotting time & INR (International Normalised Ratio) | Hu Z et al. Herb-drug interactions: a literature review. Drugs. 2005;65(9):1239-82. |
|  |  | Review |  |  | **Did not significantly alter the pharmacokinetics and pharma- codynamics** | Chen XW et al. Herb-drug interactions and mechanistic and clinical considerations. Curr Drug Metab. 2012 Jun 1;13(5):640-51. |
|  |  | Review |  |  | Alters the blood haemostasis and anticoagulation | Leite PM et al. Review on mechanisms and interactions in concomitant use of herbs and warfarin therapy. Biomed Pharmacother. 2016 Oct;83:14-21. |
|  |  | Review |  |  | Alters bleeding time | Vahabi S et al. A mini-review on herb-anesthesia drug interactions. Biomed Pharmacother. 2016 Dec;84:1885-1890. |
|  |  | Review |  |  | Increased INR, increased clotting time | Fugh-Berman A et al. Herb-drug interactions: review and assessment of report reliability. Br J Clin Pharmacol. 2001 Nov;52(5):587-95. |
|  |  | Review |  |  |  | Milić N et al. Warfarin interactions with medicinal herbs. Nat Prod Commun. 2014 Aug;9(8):1211-6. |
|  |  | Review |  |  | Recommendation- Exercise caution | Williamson EM. Interactions between herbal and conventional medicines. Expert Opin Drug Saf. 2005 Mar;4(2):355-78. |
|  |  | Review |  |  | Increases risk of bleeding | Awang DV et al. Herbal interactions with cardiovascular drugs. J Cardiovasc Nurs. 2002 Jul;16(4):64-70. |
|  |  | Clinical Trial |  |  | Suboptimal anticoagulation control | Chan HT et al. Effect of herbal consumption on time in therapeutic range of warfarin therapy in patients with atrial fibrillation. J Cardiovasc Pharmacol. 2011 Jul;58(1):87-90. |
|  |  | Review |  |  | Increase the clotting time and international normalized ratio | Chen XW et al. Clinical herbal interactions with conventional drugs: from molecules to maladies. Curr Med Chem. 2011;18(31):4836-50. |
|  |  | Review |  |  | Increase the risk of bleeding or potentiate the effects of warfarin therapy | Heck AM et al. Potential interactions between alternative therapies and warfarin. Am J Health Syst Pharm. 2000 Jul 1;57(13):1221-7; |
|  |  | Review |  |  | Causes Haemorrhage | Vaes LP et al. Interactions of warfarin with garlic, ginger, ginkgo, or ginseng: nature of the evidence. Ann Pharmacother. 2000 Dec;34(12):1478-82. |
|  |  | Clinical Trial |  |  | No reaction | Mohammed Abdul MI et al. Pharmacodynamic interaction of warfarin with cranberry but not with garlic in healthy subjects. Br J Pharmacol. 2008 Aug;154(8):1691-700. |
|  |  | Review |  |  | One case report of increased INR; no effect on warfarin pharmacokinetics or pharmacodynamics | Shi S et al. Drug interactions with herbal medicines. Clin Pharmacokinet. 2012 Feb 1;51(2):77-104. |
|  |  | Review |  |  | Decreases blood concentrations | Izzo AA et al. Interactions between herbal medicines and prescribed drugs: a systematic review. Drugs. 2001;61(15):2163-75. |
|  |  | Review |  |  | **Had no significant effect** | Choi S et al. A systematic review of the pharmacokinetic and pharmacodynamic interactions of herbal medicine with warfarin. PLoS One. 2017 Aug 10;12(8):e0182794. |
|  |  | Clinical Trial |  |  | **At recommended doses, does not affect the PD or PK** | Jiang X et al. Effect of ginkgo and ginger on the pharmacokinetics and pharmacodynamics of warfarin in healthy subjects. Br J Clin Pharmacol. 2005 Apr;59(4):425-32. |
|  |  | Review |  | Warfarin sodium | Alters the bleeding time | Miller LG. Herbal medicinals: selected clinical considerations focusing on known or potential drug-herb interactions. Arch Intern Med. 1998 Nov 9;158(20):2200-11. doi: 10.1001/archinte.158.20.2200. PMID: 9818800. |
|  |  | Review |  | Saquinavir | Decreased Maximum Plasma concentration | Hu Z et al; Herb-drug interactions: a literature review. Drugs. 2005;65(9):1239-82. |
|  |  | Review |  |  | Decreased the plasma area under the concentration-time curve (AUC) | Chen XW et al; Herb-drug interactions and mechanistic and clinical considerations. Curr Drug Metab. 2012 Jun 1;13(5):640-51. |
|  |  | Meta-analysis |  |  | Decreased saquinavir blood concentration | Izzo AA et al;. A Critical Approach to Evaluating Clinical Efficacy, Adverse Events and Drug Interactions of Herbal Remedies. Phytother Res. 2016 May;30(5):691-700. |
|  |  | Review |  |  | Decreases plasma concentration | Skalli S et al;Drug interactions with herbal medicines. Ther Drug Monit. 2007 Dec;29(6):679-86. |
|  |  | Review |  |  |  | Woodward KN. The potential impact of the use of homeopathic and herbal remedies on monitoring the safety of prescription products. Hum Exp Toxicol. 2005 May;24(5):219-33. |
|  |  | Review |  |  | Decreasing peak plasma concentration | Colalto C. Herbal interactions on absorption of drugs: Mechanisms of action and clinical risk assessment. Pharmacol Res. 2010 Sep;62(3):207-27. |
|  |  | Review |  |  | Reduce the area under the plasma concentration-time curve (AUC) and maximum concentration of saquinavir | Chen XW, Serag ES, Sneed KB, Liang J, Chew H, Pan SY, Zhou SF. Clinical herbal interactions with conventional drugs: from molecules to maladies. Curr Med Chem. 2011;18(31):4836-50. |
|  |  | Review |  |  | Decreased C max and Auc | Shi S, Klotz U. Drug interactions with herbal medicines. Clin Pharmacokinet. 2012 Feb 1;51(2):77-104. |
|  |  | Review |  |  | Sig. decrease of cmax (54%), c8h (49%) and AUC | Berginc K, Kristl A. The effect of garlic supplements and phytochemicals on the ADMET properties of drugs. Expert Opin Drug Metab Toxicol. 2012 Mar;8(3):295-310. |
|  |  | Review |  | Ritonavir and Paracetamol | Reduced Plasma Concentration | Hu Z et al; Herb-drug interactions: a literature review. Drugs. 2005;65(9):1239-82. |
|  |  | In vitro |  | Saquinavir and darunavir | Increased the uptake of the drugs | Berginc K et al; The influence of aged garlic extract on the uptake of saquinavir and darunavir into HepG2 cells and rat liver slices. Drug Metab Pharmacokinet. 2010;25(3):307-13. |
|  |  | Review |  | Chlorpropamide | Hypoglycemia | Hu Z et al; Herb-drug interactions: a literature review. Drugs. 2005;65(9):1239-82. |
|  |  | Review |  |  | Hypoglycemia | Skalli S et al; Drug interactions with herbal medicines. Ther Drug Monit. 2007 Dec;29(6):679-86. |
|  |  | Review |  |  |  | Woodward KN. The potential impact of the use of homeopathic and herbal remedies on monitoring the safety of prescription products. Hum Exp Toxicol. 2005 May;24(5):219-33. |
|  |  | Review |  |  | Cause hypoglycaemia | Chen XW et al; Clinical herbal interactions with conventional drugs: from molecules to maladies. Curr Med Chem. 2011;18(31):4836-50. |
|  |  | Review |  |  | Produces hypoglycaemia | Izzo AA et al; Interactions between herbal medicines and prescribed drugs: a systematic review. Drugs. 2001;61(15):2163-75. |
|  |  | Review |  | Atorvastatin | CYP2C9, CYP3A4 & CYP2D6 (inhibition) P-gp induction | Shaikh AS et al; Herb-drug interaction studies of herbs used in treatment of cardiovascular disorders-A narrative review of preclinical and clinical studies. Phytother Res. 2020 May;34(5):1008-1026. |
|  |  | Review |  | Alprazolam | No significant plasma change | Chen XW et al; Herb-drug interactions and mechanistic and clinical considerations. Curr Drug Metab. 2012 Jun 1;13(5):640-51. |
|  |  | Review |  |  | No effect | Shi S, Klotz U. Drug interactions with herbal medicines. Clin Pharmacokinet. 2012 Feb 1;51(2):77-104. |
|  |  | Review |  | Docetaxel | Slightly reduced the clearance | Chen XW et al; Herb-drug interactions and mechanistic and clinical considerations. Curr Drug Metab. 2012 Jun 1;13(5):640-51. |
|  |  | Review |  |  | Reduced the clearance | Yang AK et al;  Herbal interactions with anticancer drugs: mechanistic and clinical considerations. Curr Med Chem. 2010;17(16):1635-78. |
|  |  | Review |  |  | No effect | Shi S, Klotz U. Drug interactions with herbal medicines. Clin Pharmacokinet. 2012 Feb 1;51(2):77-104. |
|  |  | Review |  |  | No effect | Berginc K, Kristl A. The effect of garlic supplements and phytochemicals on the ADMET properties of drugs. Expert Opin Drug Metab Toxicol. 2012 Mar;8(3):295-310. |
|  |  | Review |  |  | Decrease docetaxel clearance. | Fasinu PS, Rapp GK. Herbal Interaction With Chemotherapeutic Drugs-A Focus on Clinically Significant Findings. Front Oncol. 2019 Dec 3;9:1356. |
|  |  | Review |  |  | **No significant modification for AUC** | Gougis P et al; Potential cytochrome P450-mediated pharmacokinetic interactions between herbs, food, and dietary supplements and cancer treatments. Crit Rev Oncol Hematol. 2021 Oct;166:103342. |
|  |  | Review |  | Acetaminophen | Did not alter the oxidative and conjugation me- tabolism | Chen XW et al; Herb-drug interactions and mechanistic and clinical considerations. Curr Drug Metab. 2012 Jun 1;13(5):640-51. |
|  |  | Review |  |  | No changes of oxidative metabolism | Berginc K, Kristl A. The effect of garlic supplements and phytochemicals on the ADMET properties of drugs. Expert Opin Drug Metab Toxicol. 2012 Mar;8(3):295-310. |
|  |  | Review |  | Ritonavir | Insignificantly altered | Chen XW et al; Herb-drug interactions and mechanistic and clinical considerations. Curr Drug Metab. 2012 Jun 1;13(5):640-51. |
|  |  | Review |  |  |  | Woodward KN. The potential impact of the use of homeopathic and herbal remedies on monitoring the safety of prescription products. Hum Exp Toxicol. 2005 May;24(5):219-33. |
|  |  | Review |  |  | Non- significantly decreased the peak plasma concentration | Colalto C. Herbal interactions on absorption of drugs: Mechanisms of action and clinical risk assessment. Pharmacol Res. 2010 Sep;62(3):207-27. |
|  |  | Review |  |  | No effect | Shi S, Klotz U. Drug interactions with herbal medicines. Clin Pharmacokinet. 2012 Feb 1;51(2):77-104. |
|  |  | Review |  |  | No sig. AUC (17% decrease) or cmax (1% decrease) changes | Berginc K, Kristl A. The effect of garlic supplements and phytochemicals on the ADMET properties of drugs. Expert Opin Drug Metab Toxicol. 2012 Mar;8(3):295-310. |
|  |  | In vitro |  |  | Concentration-dependent inhibition | Patel J et al;  In vitro interaction of the HIV protease inhibitor ritonavir with herbal constituents: changes in P-gp and CYP3A4 activity. Am J Ther. 2004 Jul-Aug;11(4):262-77. |
|  |  | Review |  | Simvastatin and pravastatin | No significant alteration | Chen XW et al; Herb-drug interactions and mechanistic and clinical considerations. Curr Drug Metab. 2012 Jun 1;13(5):640-51. |
|  |  | Review |  | Simvastatin | No effect | Shi S, Klotz U. Drug interactions with herbal medicines. Clin Pharmacokinet. 2012 Feb 1;51(2):77-104. |
|  |  | Review |  | Pravastatin | No effect | Shi S, Klotz U. Drug interactions with herbal medicines. Clin Pharmacokinet. 2012 Feb 1;51(2):77-104. |
|  |  | Review |  | Antiretroviral drugs |  | Fasinu PS et al; Clinically Relevant Pharmacokinetic Herb-drug Interactions in Antiretroviral Therapy. Curr Drug Metab. 2015;17(1):52-64. |
|  |  | Review |  |  | Significant interactions | Van den Bout-van den Beukel CJ et al; Possible drug-metabolism interactions of medicinal herbs with antiretroviral agents. Drug Metab Rev. 2006;38(3):477-514. |
|  |  | Review |  |  |  | Müller AC, Kanfer I. Potential pharmacokinetic interactions between antiretrovirals and medicinal plants used as complementary and African traditional medicines. Biopharm Drug Dispos. 2011 Nov;32(8):458-70. |
|  |  | Review |  | Aspirin or warfarin | Risk of bleeding | Agbabiaka TB et al; Concurrent Use of Prescription Drugs and Herbal Medicinal Products in Older Adults: A Systematic Review. Drugs Aging. 2017 Dec;34(12):891-905. |
|  |  | Review |  | Aspirin | Enhances the bleeding risk | Abebe W. Herbal medication: potential for adverse interactions with analgesic drugs. J Clin Pharm Ther. 2002 Dec;27(6):391-401. |
|  |  | Review |  | Neurological and Psychotic drugs |  | Wilson V, Maulik SK. Herb-Drug Interactions in Neurological Disorders: A Critical Appraisal. Curr Drug Metab. 2018;19(5):443-453. |
|  |  | Review |  | Chlorzoxazone | Increased plasma concentration of chlorzoxazone | Izzo AA et al; A Critical Approach to Evaluating Clinical Efficacy, Adverse Events and Drug Interactions of Herbal Remedies. Phytother Res. 2016 May;30(5):691-700. |
|  |  | Review |  |  | Reduces plasma concentrations | Izzo AA et al; Interactions between herbal medicines and prescribed drugs: an updated systematic review. Drugs. 2009;69(13):1777-98. |
|  |  | Review |  |  | Decreased 6-hydroxychlorzoxazone/chlorzoxazone serum ratios | Shi S, Klotz U. Drug interactions with herbal medicines. Clin Pharmacokinet. 2012 Feb 1;51(2):77-104. |
|  |  | Review |  |  | Decrease of serum 6-hydroxychlorzoxazone/ chlorzoxazone ratio | Berginc K, Kristl A. The effect of garlic supplements and phytochemicals on the ADMET properties of drugs. Expert Opin Drug Metab Toxicol. 2012 Mar;8(3):295-310. |
|  |  | Review |  | Paracetamol | Changes in paracetamol pharmacokinetics | Izzo AA et al; A Critical Approach to Evaluating Clinical Efficacy, Adverse Events and Drug Interactions of Herbal Remedies. Phytother Res. 2016 May;30(5):691-700. |
|  |  | Review |  |  | Pharmacokinetic variables of paracetamol changes | Skalli S et al; Drug interactions with herbal medicines. Ther Drug Monit. 2007 Dec;29(6):679-86. |
|  |  | Review |  |  | No effect | Shi S et al; Drug interactions with herbal medicines. Clin Pharmacokinet. 2012 Feb 1;51(2):77-104. |
|  |  | Review |  |  | Changes pharmacokinetic variables | Izzo AA et al; Interactions between herbal medicines and prescribed drugs: a systematic review. Drugs. 2001;61(15):2163-75. |
|  |  | Review |  | Chlorpropamide, fluindione, ritonavir and warfarin; |  | Izzo AA et al; Interactions between herbal medicines and prescribed drugs: an updated systematic review. Drugs. 2009;69(13):1777-98. |
|  |  | Review |  | General anesthetics | Antagonistic effect on immunosuppressing  medications | Vahabi S, Eatemadi A. Phyto-anesthetics: A mini-review on herb-anesthesia drug interactions. Biomed Pharmacother. 2016 Dec;84:1885-1890. |
|  |  | Review |  | Glibenclamide | Increases the hypoglycaemic activity, Procyclidine | Influence of Allium sativum extract on the hypoglycemic activity of glibenclamide: an approach to possible herb-drug interaction. Drug Metabol Drug Interact. 2013;28(4):225-30. |
|  |  | Review |  | Anticoagulants |  | Colalto C. Herbal interactions on absorption of drugs: Mechanisms of action and clinical risk assessment. Pharmacol Res. 2010 Sep;62(3):207-27. |
|  |  | Review |  | Coumarin anticoagulants | Affects the normal coagu- lation and platelet activity | Mousa SA. Antithrombotic effects of naturally derived products on coagulation and platelet function. Methods Mol Biol. 2010;663:229-40. |
|  |  | Review |  | Aspirin, clopidogrel | Increase the bleeding risk | Aggarwal A, Ades PA. Interactions of herbal remedies with prescription cardiovascular medications. Coron Artery Dis. 2001 Nov;12(7):581-4. |
|  |  | Review |  | Midazolam and docetaxel | Does not affect the pharmacodynamics | Goey AK et al; Relevance of in vitro and clinical data for predicting CYP3A4-mediated herb-drug interactions in cancer patients. Cancer Treat Rev. 2013 Nov;39(7):773-83. |
|  |  | Review |  | Caffeine | No effect | Shi S, Klotz U. Drug interactions with herbal medicines. Clin Pharmacokinet. 2012 Feb 1;51(2):77-104. |
|  |  | Review |  | Ciclosporin | No effect | Shi S, Klotz U. Drug interactions with herbal medicines. Clin Pharmacokinet. 2012 Feb 1;51(2):77-104. |
|  |  | Review |  | Ciclosporin A | No effect | Berginc K, Kristl A. The effect of garlic supplements and phytochemicals on the ADMET properties of drugs. Expert Opin Drug Metab Toxicol. 2012 Mar;8(3):295-310. |
|  |  | Review |  | Debrisoquine | No effect | Shi S, Klotz U. Drug interactions with herbal medicines. Clin Pharmacokinet. 2012 Feb 1;51(2):77-104. |
|  |  | Review |  | Dextromethorphan | No effect | Shi S, Klotz U. Drug interactions with herbal medicines. Clin Pharmacokinet. 2012 Feb 1;51(2):77-104. |
|  |  | Review |  | Fluindione | Decreased INR | Shi S, Klotz U. Drug interactions with herbal medicines. Clin Pharmacokinet. 2012 Feb 1;51(2):77-104. |
|  |  | Review |  | Midazolam | No effect | Shi S, Klotz U. Drug interactions with herbal medicines. Clin Pharmacokinet. 2012 Feb 1;51(2):77-104. |
|  |  | Review |  | Dextromethorphan, Alprazolam | No effect | Berginc K, Kristl A. The effect of garlic supplements and phytochemicals on the ADMET properties of drugs. Expert Opin Drug Metab Toxicol. 2012 Mar;8(3):295-310. |
|  |  | Review |  | Caffeine, debrisoquine, midazolam, | No effect | Berginc K, Kristl A. The effect of garlic supplements and phytochemicals on the ADMET properties of drugs. Expert Opin Drug Metab Toxicol. 2012 Mar;8(3):295-310. |
|  |  | In Vivo |  | Captopril | Dislodge the effect of ISO on superoxide dismutase and catalase and retained the activities of LDH and CK-MB | Asdaq SM, Inamdar MN. Pharmacodynamic interaction of captopril with garlic in isoproterenol-induced myocardial damage in rat. Phytother Res. 2010 May;24(5):720-5. |
|  |  | Review |  | HIV Drugs | Drug level changes exist | Mills E et al; Natural health product-HIV drug interactions: a systematic review. Int J STD AIDS. 2005 Mar;16(3):181-6. |
|  |  | In Vitro |  | Atorvastatin, losartan, hydrochlorothiazide, digoxin, pravastatin | Increases absorption | Berginc K, Kristl A. The mechanisms responsible for garlic - drug interactions and their in vivo relevance. Curr Drug Metab. 2013 Jan;14(1):90-101. |
|  |  | In Vivo |  | Isoniazid | Significantly altered the pharmacokinetic parameters for isoniazid | Dhamija P et al; Effect of oral administration of crude aqueous extract of garlic on pharmacokinetic parameters of isoniazid and rifampicin in rabbits. Pharmacology. 2006;77(2):100-4. |
|  |  | In Vivo |  | Rifampicin | No effect | Dhamija P et al; Effect of oral administration of crude aqueous extract of garlic on pharmacokinetic parameters of isoniazid and rifampicin in rabbits. Pharmacology. 2006;77(2):100-4. |
|  |  | In Vivo |  | Hydrochlorothiazide | Synergistic | Asdaq SM, Inamdar MN. Pharmacodynamic interaction of garlic with hydrochlorothiazide in rats. Indian J Physiol Pharmacol. 2009 Apr-Jun;53(2):127-36. |
|  |  | In Vivo |  | Dipyridamole | Reduced the oral exposure of dipyridamole | Wang Y et al; Effect of diallyl trisulfide on the pharmacokinetics of dipyridamole in rats. Arch Pharm Res. 2011 Nov;34(11):1957-64. |
|  |  | Review |  | Prostacyclin, forskolin, indometha- cin, and dipyridamole | Potentiates their effects | Ang-Lee MK et al; Herbal medicines and perioperative care. JAMA. 2001 Jul 11;286(2):208-16. |
|  |  | Review |  | Halothane | Altered the effect of halothane-induced anaesthesia in terms of induction, recovery and heart rate parameters | Bernardo J, Valentão P. Herb-drug interactions: A short review on central and peripheral nervous system drugs. Phytother Res. 2024 Apr;38(4):1903-1931. doi: 10.1002/ptr.8120. Epub 2024 Feb 15. PMID: 38358734. |
| 2 | *Curcuma longa* L. (Turmeric) | Review | Haridra | Losartan, rosuvastatin, warfarin, Clopidogrel | CYP3A4, CYP1A2, CYP2B6, CYP2C19, CYP2C9 and pgp (inhibition) | Shaikh AS et al; Herb-drug interaction studies of herbs used in treatment of cardiovascular disorders-A narrative review of preclinical and clinical studies. Phytother Res. 2020 May;34(5):1008-1026. |
|  |  | Review |  | Warfarin |  | Chen XW et al; Herb-drug interactions and mechanistic and clinical considerations. Curr Drug Metab. 2012 Jun 1;13(5):640-51. |
|  |  | Review |  |  | Increase the risk of bleeding or potentiate the effects of warfarin therapy | Heck AM et al; Potential interactions between alternative therapies and warfarin. Am J Health Syst Pharm. 2000 Jul 1;57(13):1221-7; |
|  |  | Review |  | Neurological and Psychotic drugs |  | Wilson V et al; Herb-Drug Interactions in Neurological Disorders: A Critical Appraisal. Curr Drug Metab. 2018;19(5):443-453. |
|  |  | Review |  | Anticoagulants | Recommendation- Exercise caution | Williamson EM. Interactions between herbal and conventional medicines. Expert Opin Drug Saf. 2005 Mar;4(2):355-78. |
|  |  | Review |  | Glibenclamide, glimepiride |  | Thikekar AK et al; Herb-drug interactions in diabetes mellitus: A review based on pre-clinical and clinical data. Phytother Res. 2021 Sep;35(9):4763-4781. |
|  |  | In Vitro |  | Acalabrutinib, osimertinib and olaparib | Minor to insignificant changes | Pilla Reddy V et al; Food constituent- and herb-drug interactions in oncology: Influence of quantitative modelling on Drug labelling. Br J Clin Pharmacol. 2021 Oct;87(10):3988-4000. |
|  |  | In Vitro |  | Morphine |  | Uchaipichat V. In vitro inhibitory effects of major bioactive constituents of Andrographis paniculata, Curcuma longa and Silybum marianum on human liver microsomal morphine glucuronidation: A prediction of potential herb-drug interactions arising from andrographolide, curcumin and silybin inhibition in humans. Drug Metab Pharmacokinet. 2018 Feb;33(1):67-76. |
|  |  | Review |  | Celiprolol ; midazolam | Inhibition of intestinal expression | Colalto C. Herbal interactions on absorption of drugs: Mechanisms of action and clinical risk assessment. Pharmacol Res. 2010 Sep;62(3):207-27. |
|  |  | Review |  | Talinolol | Lowers the bioavailability | Colalto C. Herbal interactions on absorption of drugs: Mechanisms of action and clinical risk assessment. Pharmacol Res. 2010 Sep;62(3):207-27. |
|  |  | In Vitro |  | Carboplatin, etoposide, or vincristine | Marked synergistic inhibitory effect against Retinoblastoma cancer cell lines | Sreenivasan S, Krishnakumar S. Synergistic Effect of Curcumin in Combination with Anticancer Agents in Human Retinoblastoma Cancer Cell Lines. Curr Eye Res. 2015;40(11):1153-65. |
|  |  | Case Report |  | Tacrolimus |  | Nayeri A et al; Acute Calcineurin Inhibitor Nephrotoxicity Secondary to Turmeric Intake: A Case Report. Transplant Proc. 2017 Jan-Feb;49(1):198-200. |
|  |  | Review |  | Aspirin | Enhances the bleeding risk | Abebe W. Herbal medication: potential for adverse interactions with analgesic drugs. J Clin Pharm Ther. 2002 Dec;27(6):391-401. |
|  |  | Clinical Trial |  | Nifedipine | No effect | Ikehata M et al; Effects of turmeric extract on the pharmacokinetics of nifedipine after a single oral administration in healthy volunteers. J Diet Suppl. 2008;5(4):401-10. |
|  |  | In Vivo |  | Norfloxacin | Mean plasma concentration of norfloxacin was significantly high | Pavithra BH et al; Modification of pharmacokinetics of norfloxacin following oral administration of curcumin in rabbits. J Vet Sci. 2009 Dec;10(4):293-7. |
|  |  | In Vivo |  | Ranitidine | Subadditive interaction. | Orona-Ortiz A et al; Mucoadhesive effect of Curcuma longa extract and curcumin decreases the ranitidine effect, but not bismuth subsalicylate on ethanol-induced ulcer model. Sci Rep. 2019 Nov 12;9(1):16622. |
|  |  | In Vivo |  | Bismuth subsalicylate | Additive relationship, which means that there is no pharmacological interaction | Orona-Ortiz A et al; Mucoadhesive effect of Curcuma longa extract and curcumin decreases the ranitidine effect, but not bismuth subsalicylate on ethanol-induced ulcer model. Sci Rep. 2019 Nov 12;9(1):16622. |
|  |  | In Vivo |  | Midazolam | Potentiate barbiturate-induced hypnosis | Ishola IO et al; Involvement of GABAergic and nitrergic systems in the anxiolytic and hypnotic effects of *Curcuma longa*: its interaction with anxiolytic-hypnotics. Drug Metab Pers Ther. 2020 Dec 29. |
|  |  | In Vitro- Clinical Trial |  | Dextromethorphan | Increases the urine metabolic ratio of DEX/DOR | Al-Jenoobi FI et al; Effect of Curcuma longa on CYP2D6- and CYP3A4-mediated metabolism of dextromethorphan in human liver microsomes and healthy human subjects. Eur J Drug Metab Pharmacokinet. 2015 Mar;40(1):61-6. |
|  |  | Review |  | Gemcitabine | Safe | Hou YN et al; Practical Application of "About Herbs" Website: Herbs and Dietary Supplement Use in Oncology Settings. Cancer J. 2019 Sep/Oct;25(5):357-366. |
|  |  | Review |  | Doxorubicin and cyclophosphamide | Interferes | Hou YN et al; Practical Application of "About Herbs" Website: Herbs and Dietary Supplement Use in Oncology Settings. Cancer J. 2019 Sep/Oct;25(5):357-366. |
|  |  | Review |  | Midazolam hamide | No significant decrease in metabolite levels | Hou YN et al; Practical Application of "About Herbs" Website: Herbs and Dietary Supplement Use in Oncology Settings. Cancer J. 2019 Sep/Oct;25(5):357-366. |
|  |  | Case Series |  | Paclitaxel | Beneficial mod- ulation of chemotherapy-related adverse effects | Kalluru H et al; Turmeric supplementation improves the quality of life and hematological parameters in breast cancer patients on paclitaxel chemotherapy: A case series. Complement Ther Clin Pract. 2020 Nov;41:101247. |
|  |  | Review |  |  | Adjuvant in chmotherapy | Lin SR et al;Natural compounds as potential adjuvants to cancer therapy: Preclinical evidence. Br J Pharmacol. 2020 Mar;177(6):1409-1423. |
|  |  | In Vivo |  | Gliclazide | Significantly greater reduction in percent blood glucose; No significant changes were observed in insulin levels, insulin resistance, and β-cell function | Vatsavai LK et al; Influence of curcumin on the pharmacodynamics and pharmacokinetics of gliclazide in animal models. J Exp Pharmacol. 2016 Nov 17;8:69-76. |
|  |  | In Vivo |  | Losartan | Enhances the plasma concentration | Liu AC et al; Pre-treatment with curcumin enhances plasma concentrations of losartan and its metabolite EXP3174 in rats. Biol Pharm Bull. 2012;35(2):145-50. |
|  |  | Review |  | Fluoxetine | Able to enhance the antidepressant effect of a sub effective dose of fluoxetine, suggesting a pharmacodynamic interaction | Bernardo J, Valentão P. Herb-drug interactions: A short review on central and peripheral nervous system drugs. Phytother Res. 2024 Apr;38(4):1903-1931. doi: 10.1002/ptr.8120. Epub 2024 Feb 15. PMID: 38358734. |
| 3 | *Zingiber officinale* ROSC. (Ginger) | Review | Nāgaram | Crizotinib | Increases concentration | Revol B et al; Pharmacokinetic herb-drug interaction between ginger and crizotinib. Br J Clin Pharmacol. 2020 Sep;86(9):1892-1893. |
|  |  | Review |  | Tacrolimus | Increases concentration | Revol B et al; Pharmacokinetic herb-drug interaction between ginger and crizotinib. Br J Clin Pharmacol. 2020 Sep;86(9):1892-1893. |
|  |  | Review |  | Nifedipine, Phenprocoumon | CYP2C9 (potent inhibition) CYP2C19, CYP3A4 (moderate inhibition) | Shaikh AS et al; Herb-drug interaction studies of herbs used in treatment of cardiovascular disorders-A narrative review of preclinical and clinical studies. Phytother Res. 2020 May;34(5):1008-1026. |
|  |  | Review |  | Warfarin | Alter the blood haemostasis and anticoagulation | Leite PM et al; Review on mechanisms and interactions in concomitant use of herbs and warfarin therapy. Biomed Pharmacother. 2016 Oct;83:14-21. |
|  |  | Review |  |  | Alteration of bleeding time | Vahabi S et al; A mini-review on herb-anesthesia drug interactions. Biomed Pharmacother. 2016 Dec;84:1885-1890. |
|  |  | Review |  |  |  | Milić N et al; Warfarin interactions with medicinal herbs. Nat Prod Commun. 2014 Aug;9(8):1211-6. |
|  |  | Review |  |  | No effect | Colalto C. Herbal interactions on absorption of drugs: Mechanisms of action and clinical risk assessment. Pharmacol Res. 2010 Sep;62(3):207-27. |
|  |  | Clinical Trial |  |  | Suboptimal anticoagulation control | Chan HT et al; Effect of herbal consumption on time in therapeutic range of warfarin therapy in patients with atrial fibrillation. J Cardiovasc Pharmacol. 2011 Jul;58(1):87-90. |
|  |  | Review |  |  | Causes Haemorrhage | Vaes LP et al; Interactions of warfarin with garlic, ginger, ginkgo, or ginseng: nature of the evidence. Ann Pharmacother. 2000 Dec;34(12):1478-82. |
|  |  | Review |  |  | Had no significant effect | Choi S et al; A systematic review of the pharmacokinetic and pharmacodynamic interactions of herbal medicine with warfarin. PLoS One. 2017 Aug 10;12(8):e0182794. |
|  |  | Clinical Trial |  |  | Increased risk of self-reported bleeding | Shalansky S et al; Risk of warfarin-related bleeding events and supratherapeutic international normalized ratios associated with complementary and alternative medicine: a longitudinal analysis. Pharmacotherapy. 2007 Sep;27(9):1237-47. |
|  |  | Review |  | Chemotherapy | Reduces side effect of chemotherapy (nausea) | Vahabi S et al; A mini-review on herb-anesthesia drug interactions. Biomed Pharmacother. 2016 Dec;84:1885-1890. |
|  |  | Review |  | Warfarin sodium | Alteration of bleeding time | Skalli S et al; Drug interactions with herbal medicines. Ther Drug Monit. 2007 Dec;29(6):679-86. |
|  |  | Review |  |  |  | Miller LG. Herbal medicinals: selected clinical considerations focusing on known or potential drug-herb interactions. Arch Intern Med. 1998 Nov 9;158(20):2200-11. |
|  |  | Review |  | Metformin, Glibenclamide |  | Thikekar AK et al; Herb-drug interactions in diabetes mellitus: A review based on pre-clinical and clinical data. Phytother Res. 2021 Sep;35(9):4763-4781. |
|  |  | In Vivo |  | Amlodipine | Influenced the Cmax, AUC0-t, and Tmax. Improves the pharmacodynamic response | Alam MA et al; Effect of Hibiscus sabdariffa and Zingiber officinale on pharmacokinetics and pharmacodynamics of amlodipine. J Pharm Pharmacol. 2021 Aug 12;73(9):1151-1160. |
|  |  | Review |  | Aspirin | Enhances the bleeding risk | Abebe W. Herbal medication: potential for adverse interactions with analgesic drugs. J Clin Pharm Ther. 2002 Dec;27(6):391-401. |
|  |  | In Vivo |  | Ciprofloxacin and isoniazid | Enhanced the penetration of ciprofloxacin and Isoniazid into the lung tissues | Nduka SO et al; Effects of Zingiber officinale on the plasma pharmacokinetics and lung penetrations of ciprofloxacin and isoniazid. Am J Ther. 2013 Sep-Oct;20(5):507-13. |
|  |  | In Vitro |  | Buprenorphine | Improve its oral bioavailability | Maharao NV et al; Inhibition of glucuronidation and oxidative metabolism of buprenorphine using GRAS compounds or dietary constituents/supplements: in vitro proof of concept. Biopharm Drug Dispos. 2017 Mar;38(2):139-154. |
|  |  | Case Study |  | Metronidazole | Significantly increased the absorption and plasma half-life, and significantly decreased the elimination rate constant and clearance | Okonta JM et al; Herb-drug interaction: a case study of effect of ginger on the pharmacokinetic of metronidazole in rabbit. Indian J Pharm Sci. 2008 Mar-Apr;70(2):230-2. |
| 4 | Glycyrrhiza glabra L. (Liquorice) | Review | Yashtimadhu | Oral and topical corticosteroids | Potentiation | Fugh-Berman A. Herb-drug interactions. Lancet. 2000 Jan 8;355(9198):134-8. |
|  |  | Review |  | Atorvastatin, simvastatin, and lovastatin | CYP2B6, CYP2C9, CYP2C19 (inhibition) CYP3A4 (induction) | Shaikh AS et al; Herb-drug interaction studies of herbs used in treatment of cardiovascular disorders-A narrative review of preclinical and clinical studies. Phytother Res. 2020 May;34(5):1008-1026. |
|  |  | Review |  | Pregnisolone | Decreases plasma clearance, and increases plasma [60] concentrations | Vahabi S et al; A mini-review on herb-anesthesia drug interactions. Biomed Pharmacother. 2016 Dec;84:1885-1890. |
|  |  | Review |  | Hydrocortisone | Potentiates of cutaneous vasoconstrictor response Hypertension, edema, hypokalemia | Vahabi Set al; A mini-review on herb-anesthesia drug interactions. Biomed Pharmacother. 2016 Dec;84:1885-1890. |
|  |  | Review |  | Oral contraceptives |  | Vahabi S et al; A mini-review on herb-anesthesia drug interactions. Biomed Pharmacother. 2016 Dec;84:1885-1890. |
|  |  | Review |  | Spironolactone | Pharmacological effect offset | Skalli S et al; Drug interactions with herbal medicines. Ther Drug Monit. 2007 Dec;29(6):679-86. |
|  |  | Review |  |  | Offset the pharmacological effect | Miller LG. Herbal medicinals: selected clinical considerations focusing on known or potential drug-herb interactions. Arch Intern Med. 1998 Nov 9;158(20):2200-11. |
|  |  | Review |  | Steroid, Oral contraceptive drugs and hypoglycaemic drugs | Recommendation- Exercise caution | Williamson EM. Interactions between herbal and conventional medicines. Expert Opin Drug Saf. 2005 Mar;4(2):355-78. |
|  |  | Review |  | Glibenclamide |  | Thikekar AK, Thomas AB, Chitlange SS. Herb-drug interactions in diabetes mellitus: A review based on pre-clinical and clinical data. Phytother Res. 2021 Sep;35(9):4763-4781. |
|  |  | Review |  | Prednisolone Oral contraceptives Digitalis Cyclosporin |  | Woodward KN. The potential impact of the use of homeopathic and herbal remedies on monitoring the safety of prescription products. Hum Exp Toxicol. 2005 May;24(5):219-33. |
|  |  | Review |  | Thiazide diuretics | Risk of hypokalaemia | Awang DV, Fugh-Berman A. Herbal interactions with cardiovascular drugs. J Cardiovasc Nurs. 2002 Jul;16(4):64-70. |
|  |  | Review |  | Digoxin | Pharmacodynamic intereference; Digoxin monitoring | Miller LG. Herbal medicinals: selected clinical considerations focusing on known or potential drug-herb interactions. Arch Intern Med. 1998 Nov 9;158(20):2200-11. |
|  |  | In Vivo |  | Celastrol | Increase the efflux ratio of celastrol | Yan G et al; Investigation of the influence of glycyrrhizin on the pharmacokinetics of celastrol in rats using LC-MS and its potential mechanism. Xenobiotica. 2017 Jul;47(7):607-613. |
|  |  | Review |  | Warfarin | Increase the risk of bleeding or potentiate the effects of warfarin therapy | Heck AM et al; Potential interactions between alternative therapies and warfarin. Am J Health Syst Pharm. 2000 Jul 1;57(13):1221-7; |
|  |  | In Vivo |  | Paclitaxel | Decrease the area under the curve and increase the total clearance | Ha Y, Wang T et al; Herb-Drug Interaction Potential of Licorice Extract and Paclitaxel: A Pharmacokinetic Study in Rats. Eur J Drug Metab Pharmacokinet. 2020 Apr;45(2):257-264. |
|  |  | In Vivo |  | Cyclosporine | Reduced bioavailability | Hou YC et al; Liquorice reduced cyclosporine bioavailability by activating P-glycoprotein and CYP 3A. Food Chem. 2012 Dec 15;135(4):2307-12. |
|  |  | In Vivo |  | Brucine | Impair the intestine absorption of brucine | Zhang M et al; Effects of licorice extracts on the pharmacokinetics of brucine in rats and its possible mechanism. Pak J Pharm Sci. 2020 Sep;33(5):1995-2002. |
|  |  | Review |  | Diltiazem, nifedipine and verapamil | Displaces serum bound cardiovascular drugs | Suroowan S et al; Herbal Medicine of the 21st Century: A Focus on the Chemistry, Pharmacokinetics and Toxicity of Five Widely Advocated Phytotherapies. Curr Top Med Chem. 2019;19(29):2718-2738. |
|  |  | Review |  | Propofol | Interactions may strongly reduce its clearance, increase its half-life, and extend its anaesthetic or toxic effects. | Bernardo J, Valentão P. Herb-drug interactions: A short review on central and peripheral nervous system drugs. Phytother Res. 2024 Apr;38(4):1903-1931. doi: 10.1002/ptr.8120. Epub 2024 Feb 15. PMID: 38358734. |
|  |  | Review |  | Midazolam | Increased first-pass metabolism of midazolam likely to occur at the intestinal wall | Bernardo J, Valentão P. Herb-drug interactions: A short review on central and peripheral nervous system drugs. Phytother Res. 2024 Apr;38(4):1903-1931. doi: 10.1002/ptr.8120. Epub 2024 Feb 15. PMID: 38358734. |
| 5 | *Trigonella foenum-graecum* L. (Fenugreek) | Review | Methika | Glipizide, Insulin and other drugs that may lower blood sugar levels | Excessive decrease of blood sugar levels | Skalli S et al; Drug interactions with herbal medicines. Ther Drug Monit. 2007 Dec;29(6):679-86. |
|  |  | Review |  | Glimepiride, gliclazide |  | Thikekar AK et al; Herb-drug interactions in diabetes mellitus: A review based on pre-clinical and clinical data. Phytother Res. 2021 Sep;35(9):4763-4781. |
|  |  | In Vivo |  | Theophylline | Decrease in Cmax and AUC0-t | Al-Jenoobi FI et al; Effects of fenugreek, garden cress, and black seed on theophylline pharmacokinetics in beagle dogs. Pharm Biol. 2015 Feb;53(2):296-300. |
|  |  | In Vivo |  | Phenytoin | Significant reduction in AUC0-∞ | Alkharfy KM et al; Effects of Lepidium sativum, Nigella sativa and Trigonella foenum-graceum on phenytoin pharmacokinetics in beagle dogs. Phytother Res. 2013 Dec;27(12):1800-4. |
|  |  | Clinical Trial |  | Warfarin | Suboptimal anticoagulation control | Chan HT et al; Effect of herbal consumption on time in therapeutic range of warfarin therapy in patients with atrial fibrillation. J Cardiovasc Pharmacol. 2011 Jul;58(1):87-90. |
|  |  | Review |  |  | Increase the risk of bleeding or potentiate the effects of warfarin therapy | Heck AM et al; Potential interactions between alternative therapies and warfarin. Am J Health Syst Pharm. 2000 Jul 1;57(13):1221-7; |
|  |  | In Vivo |  | Metoprolol tartrate | Significantly lower diastolic blood pressure | Bin Jardan YA et al; Effects of garden cress, fenugreek and black seed on the pharmacodynamics of metoprolol: an herb-drug interaction study in rats with hypertension. Pharm Biol. 2021 Dec;59(1):1088-1097. |
|  |  | In Vivo |  | Sildenafil | Significant reduction in the C max and AUC. | Al-Mohizea AM et al; Effects of Nigella sativa, Lepidium sativum and Trigonella foenum-graecum on sildenafil disposition in beagle dogs. Eur J Drug Metab Pharmacokinet. 2015 Jun;40(2):219-24. |
|  |  | In Vivo |  | Cyclosporine and carbamazepine | No statistically significant difference between pre- and post-treated | Al-Jenoobi FI et al; Pharmacokinetic interaction studies of fenugreek with CYP3A substrates cyclosporine and carbamazepine. Eur J Drug Metab Pharmacokinet. 2014 Jun;39(2):147-53. |
|  |  | Review |  | Aspirin | Enhances the bleeding risk | Abebe W. Herbal medication: potential for adverse interactions with analgesic drugs. J Clin Pharm Ther. 2002 Dec;27(6):391-401. |
|  |  | In Vitro |  | Sodium orthovanadate | Effectively controlled ocular histopathological and biochemical abnormalities associated with experimental type-1 diabetes | Preet A et al; Long-term effect of Trigonella foenum graecum and its combination with sodium orthovanadate in preventing histopathological and biochemical abnormalities in diabetic rat ocular tissues. Mol Cell Biochem. 2006 Sep;289(1-2):137-47. |
|  |  | In Vivo |  | Losartan | Interaction takes place | Ahad A et al; Potential pharmacodynamic and pharmacokinetic interactions of *Nigella Sativa* and *Trigonella Foenum-graecum* with losartan in L-NAME induced hypertensive rats. Saudi J Biol Sci. 2020 Oct;27(10):2544-2550. |
|  |  | In Vivo |  | Clopidogrel | Caused an increase in clopidogrel Cmax | Alkharfy K et al; Clopidogrel-Herb Interactions: A Pharmacokinetic and Pharmacodynamic Assessment in a Rat Model. Curr Drug Metab. 2021 Oct 29. |
| 6 | *Valeriana officinalis* L. (Valerian) | Review | Tagaram | Neurological and Psychotic drugs |  | Wilson V et al; Herb-Drug Interactions in Neurological Disorders: A Critical Appraisal. Curr Drug Metab. 2018;19(5):443-453. |
|  |  | Review |  | Barbiturates | Excessive sedation | Skalli S et al; Drug interactions with herbal medicines. Ther Drug Monit. 2007 Dec;29(6):679-86. |
|  |  | Review |  | Central nervous system depressants | Increased drugs effect | Skalli S et al; Drug interactions with herbal medicines. Ther Drug Monit. 2007 Dec;29(6):679-86. |
|  |  | Review |  | Tamoxifen, anti cancer drugs | Recommendation- Exercise caution | Williamson EM. Interactions between herbal and conventional medicines. Expert Opin Drug Saf. 2005 Mar;4(2):355-78. |
|  |  | Review |  | Benzodiazepines | Positively modu- late GABAA channels | Colalto C. Herbal interactions on absorption of drugs: Mechanisms of action and clinical risk assessment. Pharmacol Res. 2010 Sep;62(3):207-27. |
|  |  | Review |  | Digoxin | Inhibits the transport | Colalto C. Herbal interactions on absorption of drugs: Mechanisms of action and clinical risk assessment. Pharmacol Res. 2010 Sep;62(3):207-27. |
|  |  | Review |  | Warfarin, hepatotoxically acting medicaments, MAOI inhibitors, phenelzin sulphate, or phenytoin | Decrease or completely eliminate the therapeutic effect of the administered drugs | Tůmová L. Interakce mezi lécivými rostlinami a lécivy [Interactions between herbal medicines and drugs]. Ceska Slov Farm. 2000 Jul;49(4):162-7. Czech. |
|  |  | Case Study |  | Lorazepam | Adverse effects - Case report | Carrasco MC et al;  Interactions of Valeriana officinalis L. and Passiflora incarnata L. in a patient treated with lorazepam. Phytother Res. 2009 Dec;23(12):1795-6. |
|  |  | Review |  | Opioid analgesics | Increased central nervous system (CNS) depression | Abebe W. Herbal medication: potential for adverse interactions with analgesic drugs. J Clin Pharm Ther. 2002 Dec;27(6):391-401. |
|  |  | Review |  | Barbiturates, midazolam | Increases the sedative effect | Ang-Lee MK et al; Herbal medicines and perioperative care. JAMA. 2001 Jul 11;286(2):208-16. |
|  |  | In Vivo |  | Dexamethasone & ketoconazole |  | Bogacz A et al; The influence of standardized Valeriana officinalis extract on the CYP3A1 gene expression by nuclear receptors in in vivo model. Biomed Res Int. 2014;2014:819093. |
| 7 | *Carica papaya* L. (Papaya) | Review | Eraṇḍa karkaṭi | Warfarin | ↑ INR | Fugh-Berman A et al; Herb-drug interactions: review and assessment of report reliability. Br J Clin Pharmacol. 2001 Nov; 52(5):587-95. |
|  |  | Review |  |  | Anti coagulant effect was potentiated | Awang DV et al;Herbal interactions with cardiovascular drugs. J Cardiovasc Nurs. 2002 Jul;16(4):64-70. |
|  |  | In Vitro |  | Digoxin | Significantly inhibit p-gp | Oga EF.et al; P-glycoprotein mediated efflux in Caco-2 cell monolayers: the influence of herbals on digoxin transport. J Ethnopharmacol. 2012 Dec 18;144(3):612-7. |
|  |  | Ex Vivo and In Vivo |  |  | Increased the mean digoxin apparent permeability in the mucosal-to-serosal direction | Oga EF et al; Ex vivo and in vivo investigations of the effects of extracts of Vernonia amygdalina, Carica papaya and Tapinanthus sessilifolius on digoxin transport and pharmacokinetics: assessing the significance on rat intestinal P-glycoprotein efflux. Drug Metab Pharmacokinet. 2013;28(4):314-20. |
|  |  | Clinical Trial |  | Warfarin | Suboptimal anticoagulation control | Chan HT et al; Effect of herbal consumption on time in therapeutic range of warfarin therapy in patients with atrial fibrillation. J Cardiovasc Pharmacol. 2011 Jul;58(1):87-90. |
|  |  | In Vivo |  | Amiodarone | Increase the drug bioavailability. | Rodrigues M et al; Herb-drug pharmacokinetic interaction between carica papaya extract and amiodarone in rats. J Pharm Pharm Sci. 2014;17(3):302-15. |
|  |  | Review |  | Metformin, glimepiride, digoxin, ciprofloxacin, and artemisinin | Unfavourable interactions | Lim XY et al; *Carica papaya* L. Leaf: A Systematic Scoping Review on Biological Safety and Herb-Drug Interactions. Evid Based Complement Alternat Med. 2021 May 7;2021:5511221. |
| 8 | *Piper nigrum* L. and P. *longum* L. (Pepper) | Review | Pippali; maricam | Phenytoin, propranolol and theophylline | Increased the AUC | Hu Z et al; Herb-drug interactions: a literature review. Drugs. 2005;65(9):1239-82. |
|  |  | Review |  | Rifamipicin | Increased plasma concentrations  in TB patients | Hu Z et al; Herb-drug interactions: a literature review. Drugs. 2005;65(9):1239-82. |
|  |  | Review |  | Propranolol | Increased the Cmax and AUC | Chen XW et al; Herb-drug interactions and mechanistic and clinical considerations. Curr Drug Metab. 2012 Jun 1;13(5):640-51. |
|  |  | Review |  | Antiretroviral drugs |  | Fasinu PS et al; Clinically Relevant Pharmacokinetic Herb-drug Interactions in Antiretroviral Therapy. Curr Drug Metab. 2015;17(1):52-64. |
|  |  | Review |  | Neurological and Psychotic drugs |  | Wilson V et al; Herb-Drug Interactions in Neurological Disorders: A Critical Appraisal. Curr Drug Metab. 2018;19(5):443-453. |
|  |  | In Vivo |  | Docetaxel | Potential enhanced bioavailability of not only docetaxel but also Piperine | Chow MSS et al; Non-linear pharmacokinetics of piperine and its herb-drug interactions with docetaxel in Sprague-Dawley rats. J Pharm Biomed Anal. 2016 Sep 5;128:286-293. |
| 9 | Piper longum L. (Long pepper) |  | Pippali | Verapamil, Digoxin, Propranolol | CYP3A4, CYP2D6 and CYP1A2 (inhibition) |  |
|  |  | In Vivo |  | Oxytetracycline | Reduced loading and maintenance dose | Singh M et al; Alteration of pharmacokinetics of oxytetracycline following oral administration of Piper longum in hens. J Vet Sci. 2005 Sep;6(3):197-200. |
|  |  | In Vitro, In Vivo |  | Docetaxel | Significantly induces activity against taxane-resistant prostate tumor. | Li C et al; Enhanced anti-tumor efficacy and mechanisms associated with docetaxel-piperine combination- *in vitro* and *in vivo* investigation using a taxane-resistant prostate cancer model. Oncotarget. 2017 Dec 14;9(3):3338-3352. |
| 10 | *Piper nigrum* L. (Pepper) | Review | Marica | Phenytoin | Affects the bioavailability | Colalto C. Herbal interactions on absorption of drugs: Mechanisms of action and clinical risk assessment. Pharmacol Res. 2010 Sep;62(3):207-27. |
|  |  | Review |  | Rifampicin, nimesulide, propanolol and theophylline | Increases the bioavailability | Colalto C. Herbal interactions on absorption of drugs: Mechanisms of action and clinical risk assessment. Pharmacol Res. 2010 Sep;62(3):207-27. |
|  |  | In Vivo |  | Warfarin | Reduce the plasma concentration and anticoagulation of warfarin | Zayed A et al; Piperine Alters the Pharmacokinetics and Anticoagulation of Warfarin in Rats. J Exp Pharmacol. 2020 Jun 19;12:169-179. |
|  |  | Review |  | Domperidone | There is a significant increase in C_max_, AUC and t_1/2_ parameters of domperidone. This pharmacokinetic interaction enhances the oral bioavailability of domperidone due to inhibition of P-gp and CYP3A1 across the rat intestine and liver, and due to a decrease in the clearance and elimination rate constant mediated by inhibition of hepatic CYP3A4 | Bernardo J, Valentão P. Herb-drug interactions: A short review on central and peripheral nervous system drugs. Phytother Res. 2024 Apr;38(4):1903-1931. doi: 10.1002/ptr.8120. Epub 2024 Feb 15. PMID: 38358734. |
| 11 | *Andrographis paniculata* (BURM.F.)WALLICH EX NEES. (Kariyat) | In Vivo | Kirātatikta | Theophylline | Clearance of theophylline was significantly increased and the area under concentration-time curve (AUC) was reduced | TH. Herb-drug interaction of Andrographis paniculata extract and andrographolide on the pharmacokinetics of theophylline in rats. Chem Biol Interact. 2010 Mar 30;184(3):458-65. |
|  |  | Review |  | Tolbutamide, Gliclazide, Glyburide |  | Thikekar AK et al; Herb-drug interactions in diabetes mellitus: A review based on pre-clinical and clinical data. Phytother Res. 2021 Sep;35(9):4763-4781. |
|  |  | In Vitro |  | Morphine |  | Uchaipichat V. In vitro inhibitory effects of major bioactive constituents of Andrographis paniculata, Curcuma longa and Silybum marianum on human liver microsomal morphine glucuronidation: A prediction of potential herb-drug interactions arising from andrographolide, curcumin and silybin inhibition in humans. Drug Metab Pharmacokinet. 2018 Feb;33(1):67-76. |
|  |  | Review |  | Glibenclamide, glimepiride, glipizide, nateglinide, rosiglitazone, pioglitazone, repaglinide |  | Rehman SU et al; Interactions between herbs and antidiabetics: an overview of the mechanisms, evidence, importance, and management. Arch Pharm Res. 2015 Jul;38(7):1281-98. |
|  |  | In Vivo |  | Nabumetone |  | Mahadik K. Pharmacokinetic and Pharmacodynamic Interaction of Andrographolide and Standardized Extract of Andrographis paniculata (Nees) with Nabumetone in Wistar Rats. Phytother Res. 2017 Jan;31(1):75-80. |
|  |  | In Vivo |  | Naproxen | Decreased systemic exposure level | Balap A et al; Herb-drug interaction of Andrographis paniculata (Nees) extract and andrographolide on pharmacokinetic and pharmacodynamic of naproxen in rats. J Ethnopharmacol. 2017 Jan 4;195:214-221. |
|  |  | Review |  | Propofol | Interactions may strongly reduce its clearance, increase its half-life, and extend its anaesthetic or toxic effects. | Bernardo J, Valentão P. Herb-drug interactions: A short review on central and peripheral nervous system drugs. Phytother Res. 2024 Apr;38(4):1903-1931. doi: 10.1002/ptr.8120. Epub 2024 Feb 15. PMID: 38358734. |
|  |  | Review |  | Midazolam | Pre-treatment with *A. paniculata* did not change pharmacokinetic and pharmacodynamic parameters | Bernardo J, Valentão P. Herb-drug interactions: A short review on central and peripheral nervous system drugs. Phytother Res. 2024 Apr;38(4):1903-1931. doi: 10.1002/ptr.8120. Epub 2024 Feb 15. PMID: 38358734. |
| 12 | *Aloe vera* L. (Aloe) | Review | Kumāri | Chlorpropamide | Less glycosuria | Vahabi S et al; Phyto-anesthetics: A mini-review on herb-anesthesia drug interactions. Biomed Pharmacother. 2016 Dec;84:1885-1890. |
|  |  | Review |  | Glibenclamide |  | Thikekar AK et al; Herb-drug interactions in diabetes mellitus: A review based on pre-clinical and clinical data. Phytother Res. 2021 Sep;35(9):4763-4781. |
|  |  | Review |  | Thiazide diuretics | Enhances reduction in serum potassium | Awang DV et al; Herbal interactions with cardiovascular drugs. J Cardiovasc Nurs. 2002 Jul;16(4):64-70. |
|  |  | Review |  | Pioglitazone, repaglinide | Enhancement in adipose tissue insulin signaling pathway | Rehman SU et al; Interactions between herbs and antidiabetics: an overview of the mechanisms, evidence, importance, and management. Arch Pharm Res. 2015 Jul;38(7):1281-98. |
|  |  | In Vitro |  | Cimetidine | Did not inhibit the efflux of cimetidine, | Carien B et al; Modulation of drug efflux by aloe materials: An In Vitro investigation across rat intestinal tissue. Pharmacogn Mag. 2013 Oct;9(Suppl 1):S44-8. |
|  |  | Review |  | Sevoflurane | Synergistic effect on antiplatelet effects | Lee A et al; Possible interaction between sevoflurane and Aloe vera. Ann Pharmacother. 2004 Oct;38(10):1651-4. |
| 13 | *Withania somnifera* DUNAL (Winter cherry) | Review | Aśvagandha | Barbiturates and benzodiazepines | Potentiates sedative effect | Williamson EM. Interactions between herbal and conventional medicines. Expert Opin Drug Saf. 2005 Mar;4(2):355-78. |
|  |  | Review |  | Glimepiride |  | Thikekar AK et al; Herb-drug interactions in diabetes mellitus: A review based on pre-clinical and clinical data. Phytother Res. 2021 Sep;35(9):4763-4781. |
|  |  | Review |  | Oxaliplatin | Enhanced the effects | Hou YN et al; Practical Application of "About Herbs" Website: Herbs and Dietary Supplement Use in Oncology Settings. Cancer J. 2019 Sep/Oct;25(5):357-366. |
|  |  | Review |  | Paclitaxel | Reversed paclitaxel-induced neutropenia | Hou YN et al; Practical Application of "About Herbs" Website: Herbs and Dietary Supplement Use in Oncology Settings. Cancer J. 2019 Sep/Oct;25(5):357-366. |
|  |  | Review |  | Anticonvulsants, barbiturates, and benzodiazepines | Increases the sedative effect | Hou YN et al; Practical Application of "About Herbs" Website: Herbs and Dietary Supplement Use in Oncology Settings. Cancer J. 2019 Sep/Oct;25(5):357-366. |
|  |  | In Vitro |  | Digoxin | Interfered with serum digoxin measurements | Dasgupta A et al; Effect of Indian Ayurvedic medicine Ashwagandha on measurement of serum digoxin and 11 commonly monitored drugs using immunoassays: study of protein binding and interaction with Digibind. Arch Pathol Lab Med. 2007 Aug;131(8):1298-303. |
| 14 | *Areca catechu* L. (Areca nut) | Review | Kramuka/poogaphala | Neuroleptic drugs |  | Fugh-Berman A. Herb-drug interactions. Lancet. 2000 Jan 8;355(9198):134-8. |
|  |  | Review |  | Procyclidine | Rigidity, bradykinesia and jaw tremors | Izzo AA et al; A Critical Approach to Evaluating Clinical Efficacy, Adverse Events and Drug Interactions of Herbal Remedies. Phytother Res. 2016 May;30(5):691-700. |
|  |  | Review |  | Fluphenazine; Procyclidine | Rigidity, bradykinesia, jaw tremor | Fugh-Berman A et al; Herb-drug interactions: review and assessment of report reliability. Br J Clin Pharmacol. 2001 Nov;52(5):587-95. |
|  |  | Review |  | Flupenthixol | Stiffness, tremor, akathisia | Fugh-Berman A et al; Herb-drug interactions: review and assessment of report reliability. Br J Clin Pharmacol. 2001 Nov;52(5):587-95. |
|  |  | Clinical Trial |  | Tacrolimus | Dose-adjusted blood trough levels of tacrolimus is low | Chen WY et al; Betel Nut Chewing Is Associated With Reduced Tacrolimus Concentration in Taiwanese Liver Transplant Recipients. Transplant Proc. 2017 Mar;49(2):326-329. |
|  |  | Review |  | Procyclidine | Antagonism pharmacodynamic interaction – Anticholinergic activity | Bernardo J, Valentão P. Herb-drug interactions: A short review on central and peripheral nervous system drugs. Phytother Res. 2024 Apr;38(4):1903-1931. doi: 10.1002/ptr.8120. Epub 2024 Feb 15. PMID: 38358734. |
| 15 | *Gymnema sylvestre* R.BR. (Gymnema) | Review | Ajaśṛṅgī | Glimepiride, glibenclamide, gliclazide, metformin, sitagliptin | Inhibition of CYP1A2, CYP 3A4, CYP 2C9 | Thikekar AK et al; Herb-drug interactions in diabetes mellitus: A review based on pre-clinical and clinical data. Phytother Res. 2021 Sep;35(9):4763-4781. |
|  |  | In Vivo |  | Glimepiride | Beneficial pharmacodynamic interactions whereas no major alterations in the pharmacokinetics parameters of GLM and GMG were observed | Effects of Gymnema sylvestre extract on the pharmacokinetics and pharmacodynamics of glimepiride in streptozotocin induced diabetic rats. Chem Biol Interact. 2016 Feb 5;245:30-8. |
|  |  | In Vivo |  | Tolbutamide | Clearance rate was significantly decreased; Cmax, and AUC0-24 | Vaghela M et al; In vivo pharmacokinetic interaction by ethanolic extract of Gymnema sylvestre with CYP2C9 (Tolbutamide), CYP3A4 (Amlodipine) and CYP1A2 (Phenacetin) in rats. Chem Biol Interact. 2017 Dec 25;278:141-151. |
|  |  | In Vivo |  | Amlodipine | PK parameters were not significantly affected | Vaghela M et al; In vivo pharmacokinetic interaction by ethanolic extract of Gymnema sylvestre with CYP2C9 (Tolbutamide), CYP3A4 (Amlodipine) and CYP1A2 (Phenacetin) in rats. Chem Biol Interact. 2017 Dec 25;278:141-151. |
|  |  | In Vivo |  | Phenacetin | Area under the plasma concentration-time curve , significantly increased | Vaghela M et al; In vivo pharmacokinetic interaction by ethanolic extract of Gymnema sylvestre with CYP2C9 (Tolbutamide), CYP3A4 (Amlodipine) and CYP1A2 (Phenacetin) in rats. Chem Biol Interact. 2017 Dec 25;278:141-151. |
| 16 | *Nigella sativa* L. (Fennel flower) | In Vivo | Kāravī | Phenytoin | More drastic on drug elimination and to a lesser extent on its volume of distribution at steady state (Vss ) with a consequent reduction in systemic exposure measured by area under the curve (AUC0-∞ ) | Alkharfy KM et al; Effects of Lepidium sativum, Nigella sativa and Trigonella foenum-graceum on phenytoin pharmacokinetics in beagle dogs. Phytother Res. 2013 Dec;27(12):1800-4. |
|  |  | In Vivo |  | Metoprolol tartrate | Significantly lower diastolic blood pressure | Bin Jardan YA et al; Effects of garden cress, fenugreek and black seed on the pharmacodynamics of metoprolol: an herb-drug interaction study in rats with hypertension. Pharm Biol. 2021 Dec;59(1):1088-1097. |
|  |  | In Vivo |  | Sildenafil | Reduction of AUC0-∞, C max and t 1/2 | Al-Mohizea AM et al; Effects of Nigella sativa, Lepidium sativum and Trigonella foenum-graecum on sildenafil disposition in beagle dogs. Eur J Drug Metab Pharmacokinet. 2015 Jun;40(2):219-24. |
|  |  | In Vivo |  | Losartan | Interaction takes place | Ahad A et al; Potential pharmacodynamic and pharmacokinetic interactions of *Nigella Sativa* and *Trigonella Foenum-graecum* with losartan in L-NAME induced hypertensive rats. Saudi J Biol Sci. 2020 Oct;27(10):2544-2550. |
|  |  | In Vivo |  | Clopidogrel | Caused an increase in clopidogrel Cmax | Alkharfy K et al; Clopidogrel-Herb Interactions: A Pharmacokinetic and Pharmacodynamic Assessment in a Rat Model. Curr Drug Metab. 2021 Oct 29. |
|  |  | In Vivo |  | Prednisolone | Decreased prednisolone Cmax and AUC0-last in rats indicating that there is a herb-drug interaction | Abutaima R, Al-Ebini Y, Alkofahi A, Alshishani A, Thiab S, Alagammai KC, Khalid M. In vivo assessment of black seed oil single dose on prednisolone pharmacokinetics. J Pharm Pharmacol. 2024 Jan 6;76(1):57-63. doi: 10.1093/jpp/rgad110. PMID: 37978932. |
| 17 | *Moringa oleifera* LAM. (Drumstick) | Review | Śigru | Metformin |  | Thikekar AK et al; Herb-drug interactions in diabetes mellitus: A review based on pre-clinical and clinical data. Phytother Res. 2021 Sep;35(9):4763-4781. |
|  |  | Clinical Trial |  | Amodiaquine | Alters the pharmacokinetics | Olawoye OS et al; Moringa oleifera leaf powder alters the pharmacokinetics of amodiaquine in healthy human volunteers. J Clin Pharm Ther. 2018 Oct;43(5):626-632. |
|  |  | Clinical Trial |  | Nevirapine | Did not significantly alter the steady-state PK of nevirapine | Monera-Penduka TG et al; Effect of *Moringa oleifera* Lam. leaf powder on the pharmacokinetics of nevirapine in HIV-infected adults: a one sequence cross-over study. AIDS Res Ther. 2017 Mar 14;14:12. |
|  |  | In Vivo |  | Sitagliptin | Progressive decrease in anti-hyperglycaemic effect | Olurishe C et al; Chronic administration of ethanol leaf extract of Moringa oleifera Lam. (Moringaceae) may compromise glycaemic efficacy of Sitagliptin with no significant effect in retinopathy in a diabetic rat model. J Ethnopharmacol. 2016 Dec 24;194:895-903. |
| 18 | *Lepidium sativum* L. (Common cress) | In Vivo | Jivanti | Phenytoin | Modest increase in maximum observed concentration (Cmax ) and terminal half-life (T1/2λ ) of phenytoin with a reduction in clearance by 33% | Alkharfy KM et al; Effects of Lepidium sativum, Nigella sativa and Trigonella foenum-graceum on phenytoin pharmacokinetics in beagle dogs. Phytother Res. 2013 Dec;27(12):1800-4. |
|  |  | In Vivo |  | Metoprolol tartrate | Significantly lower diastolic blood pressure | Bin Jardan YA et al; Effects of garden cress, fenugreek and black seed on the pharmacodynamics of metoprolol: an herb-drug interaction study in rats with hypertension. Pharm Biol. 2021 Dec;59(1):1088-1097. |
|  |  | In Vivo |  | Sildenafil | Significant reduction in the C max and AUC. | Al-Mohizea AM et al; Effects of Nigella sativa, Lepidium sativum and Trigonella foenum-graecum on sildenafil disposition in beagle dogs. Eur J Drug Metab Pharmacokinet. 2015 Jun;40(2):219-24. |
|  |  | In Vivo |  | Clopidogrel | No change | Alkharfy K et al; Clopidogrel-Herb Interactions: A Pharmacokinetic and Pharmacodynamic Assessment in a Rat Model. Curr Drug Metab. 2021 Oct 29. |
| 19 | *Convolvulus pluricaulis* CHOIS. (Type of Morning Glory) | Review | Śaṅkhapuṣpi | Phenytoin | Decreased concentration | Fugh-Berman A. Herb-drug interactions. Lancet. 2000 Jan 8;355(9198):134-8. |
|  |  | Review |  |  | Decreased phenytoin concentration loss of control of [53] seizures | Vahabi S et al; A mini-review on herb-anesthesia drug interactions. Biomed Pharmacother. 2016 Dec;84:1885-1890. |
|  |  | Review |  |  | Decreases the level and the efficacy | Miller LG. Herbal medicinals: selected clinical considerations focusing on known or potential drug-herb interactions. Arch Intern Med. 1998 Nov 9;158(20):2200-11. |
| 20 | *Momordica charantia* L. (Bitter gourd) | Review | Kāravella | Diabetes mellitus drugs | Blood glucose level effect | Skalli S et al; Drug interactions with herbal medicines. Ther Drug Monit. 2007 Dec;29(6):679-86. |
|  |  | Review |  | Rosiglitazone, Glibenclamide, Metformin |  | Thikekar AK et al; Herb-drug interactions in diabetes mellitus: A review based on pre-clinical and clinical data. Phytother Res. 2021 Sep;35(9):4763-4781. |
|  |  | In Vivo |  | Glibenclamide | Greater glycemic improvement than GLB monotherapy. | Abdel-Rahman RF et al; Molecular and biochemical monitoring of the possible herb-drug interaction between *Momordica charantia* extract and glibenclamide in diabetic rats. Saudi Pharm J. 2019 Sep;27(6):803-816. |
| 21 | *Vitis vinifera* L. (Grapes/ Grape seed ) | Review | Drākṣa | Anti cancer drugs | Recommendation- Exercise caution | Williamson EM. Interactions between herbal and conventional medicines. Expert Opin Drug Saf. 2005 Mar;4(2):355-78. |
|  |  | In Vivo |  | Imatinib | Affects the pharmacokinetics | Darweesh RS et al; The effect of grape seed and green tea extracts on the pharmacokinetics of imatinib and its main metabolite, N-desmethyl imatinib, in rats. BMC Pharmacol Toxicol. 2020 Nov 16;21(1):77. |
|  |  | In Vitro |  | Midazolam | Alters the pharmacokinetics of midazolam | Nishikawa M et al; Effects of continuous ingestion of green tea or grape seed extracts on the pharmacokinetics of midazolam. Drug Metab Pharmacokinet. 2004 Aug;19(4):280-9. |
| 22 | *Eugenia jambolana* LAM. (Java plum) | Review | Jambū | Sitagliptin | Significant reduction in Cmax and in AUC0-24 | Thikekar AK et al; Herb-drug interactions in diabetes mellitus: A review based on pre-clinical and clinical data. Phytother Res. 2021 Sep;35(9):4763-4781. |
|  |  | In Vivo |  |  | Reduced systemic exposure of SITA without compromising on its antihyperglycemic activity and improvement in conditions associated with diabetes. | Vora A et al; *Eugenia jambolana* extract reduces the systemic exposure of Sitagliptin and improves conditions associated with diabetes: A pharmacokinetic and a pharmacodynamic herb-drug interaction study. J Tradit Complement Med. 2018 Oct 3;9(4):364-371. |
|  |  | In Vitro |  | Diclofenac, dextromethorphan |  | Chinni S et al; Effect of crude extract of Eugenia jambolana Lam. on human cytochrome P450 enzymes. Phytother Res. 2014 Nov; 28(11):1731-4. |
|  |  | Review |  | Nateglinide, Glyburide, Glimepiride, Gliclazide, Glipizide, and Rosiglitazone |  | Thikekar AK et al; Herb-drug interactions in diabetes mellitus: A review based on pre-clinical and clinical data. Phytother Res. 2021 Sep;35(9):4763-4781. |
| 23 | *Ocimum gratissimum* L. (Basil) | In Vitro | Surasa | Ampicillin | Synergistic interaction against clinical isolates of E. coli and P. mirabilis | Nweze EI et al; Justification for the use of Ocimum gratissimum L in herbal medicine and its interaction with disc antibiotics. BMC Complement Altern Med. 2009 Sep 28;9:37. |
|  |  | In Vitro |  | Septrin | Synergistic against the clinical isolate of E. coli | Nweze EI et al; Justification for the use of Ocimum gratissimum L in herbal medicine and its interaction with disc antibiotics. BMC Complement Altern Med. 2009 Sep 28;9:37. |
|  |  | In Vitro |  | Ketoconazole and nystatin. | Synergistic against C. albicans | Nweze EI et al; Justification for the use of Ocimum gratissimum L in herbal medicine and its interaction with disc antibiotics. BMC Complement Altern Med. 2009 Sep 28;9:37. |
| 24 | Cannabis (Marijuana) | Review | Gañjika | Methylenedioxymethamphetamine | Modulator effects of body temperature, conditioned reinforcement, and presumed neurotoxicity | Schulz S. MDMA & cannabis: a mini-review of cognitive, behavioral, and neurobiological effects of co-consumption. Curr Drug Abuse Rev. 2011 Jun;4(2):81-6. |
|  |  | Review |  | Irinotecan | No significant modification of AUC for irinotecan and its active metabolite SN-38 | Gougis P et al; Potential cytochrome P450-mediated pharmacokinetic interactions between herbs, food, and dietary supplements and cancer treatments. Crit Rev Oncol Hematol. 2021 Oct;166:103342. |
|  |  | Review |  | Docetaxel | No significant modification of AUC | Gougis P et al; Potential cytochrome P450-mediated pharmacokinetic interactions between herbs, food, and dietary supplements and cancer treatments. Crit Rev Oncol Hematol. 2021 Oct;166:103342. |
|  |  | Review |  | Morphine and oxycodone | Inhaled cannabis potentiates analgesia of opioids, not due to pharmacokinetic influences but via unknown pharmacodynamic interactions | Bernardo J, Valentão P. Herb-drug interactions: A short review on central and peripheral nervous system drugs. Phytother Res. 2024 Apr;38(4):1903-1931. doi: 10.1002/ptr.8120. Epub 2024 Feb 15. PMID: 38358734. |
| 25 | *Punica granatum* L. (Pomegranate) | In Vitro | Dāḍima | Novobiocin | Significantly enhanced the activity | Phatthalung PN et al; Thai ethnomedicinal plants as resistant modifying agents for combating Acinetobacter baumannii infections. BMC Complement Altern Med. 2012 Apr 26;12:56. |
|  |  | Review |  | Simvastatin | Inhibitory effect up to 59% | Rafieian-Kopaei M. Herbs with anti-lipid effects and their interactions with statins as a chemical anti- hyperlipidemia group drugs: A systematic review. ARYA Atheroscler. 2015 Jul;11(4):244-51. |
|  |  | In Vivo |  | Warfarin | Enhanced the anticoagulant activity | Alnaqeeb M et al; Critical pharmacokinetic and pharmacodynamic drug-herb interactions in rats between warfarin and pomegranate peel or guava leaves extracts. BMC Complement Altern Med. 2019 Jan 24;19(1):29. |
|  |  | Review |  | Citalopram | The chronic administration of a combination of subtherapeutic doses of citalopram and a liofylized aqueous extract of P. granatum induced an antidepressant-like effect | González-Trujano ME, Ventura-Martínez R, Silveira D, Déciga-Campos M. Editorial: Pharmacological interaction between drugs and medicinal plants, Volume II. Front Pharmacol. 2024 Jan 30;15:1372366. doi: 10.3389/fphar.2024.1372366. PMID: 38352146; PMCID: PMC10861793. |
| 26 | *Dioscorea bulbifera* L. (Acom) | In Vitro | Vārāhikanda | Doxorubicin | Delays the excretion; aggravates doxorubicin-induced cardiotoxicity and nephrotoxicity | Qu X et al; *Dioscorea bulbifera L.* delays the excretion of doxorubicin and aggravates doxorubicin-induced cardiotoxicity and nephrotoxicity by inhibiting the expression of P-glycoprotein in mice liver and kidney. Xenobiotica. 2019 Sep;49(9):1116-1125. |
|  |  | In Vitro |  | Pirarubicin | Increases the cardio toxicity | Sun LR et al; Extract from Dioscorea bulbifera L. rhizomes aggravate pirarubicin-induced cardiotoxicity by inhibiting the expression of P-glycoprotein and multidrug resistance-associated protein 2 in the mouse liver. Sci Rep. 2021 Oct 5;11(1):19720. |
| 27 | *Coleus forskohlii* (POIR.) BRIQ (Coleus) | In Vivo | Hribera | Tolbutamide | Dose-dependently attenuated both the hypoglycemic action and plasma concentration | Yokotani K et al; Coleus forskohlii extract attenuates the hypoglycemic effect of tolbutamide in vivo via a hepatic cytochrome P450-mediated mechanism. Shokuhin Eiseigaku Zasshi. 2014;55(2):73-8. |
|  |  | In Vivo. In Vitro |  | Warfarin | Attenuates the anticoagulant action | Yokotani K et al; Hepatic cytochrome P450 mediates interaction between warfarin and Coleus forskohlii extract in vivo and in vitro. J Pharm Pharmacol. 2012 Dec;64(12):1793-801. |
| 28 | *Cuminum cyminum* L. (cumin) | In Vitro | Jiraka | Rifampicin | Significant enhancement of RIF levels in plasma | Sachin BS et al; Herbal modulation of drug bioavailability: enhancement of rifampicin levels in plasma by herbal products and a flavonoid glycoside derived from Cuminum cyminum. Phytother Res. 2007 Feb;21(2):157-63. |
|  |  | In Vivo | Jiraka | Glyburide | Significantly lowered elevated glucose without causing hypoglycaemia and lipid- renal parameters | Kaur G et al; Pharmacodynamic interaction of cumin seeds (Cuminum cyminum L.) with glyburide in diabetes. J Complement Integr Med. 2019 Jul 26;16(4): |
| 29 | *Cinnamomum cassia* BLUME (cinnamaldehyde) (Cinnamon) | In Vitro | Varāṅgam | 5-fluorouracil (5-FU) and oxaliplatin (OXA) | Promising candidate as an adjuvant | Yu C et al; Cinnamaldehyde/chemotherapeutic agents interaction and drug-metabolizing genes in colorectal cancer. Mol Med Rep. 2014 Feb;9(2):669-76. |
|  |  | In Vivo |  | Pioglitazone | Enhanced the bioavailability | Mamindla S et al; Effect of Cinnamomum cassia on the Pharmacokinetics and Pharmacodynamics of Pioglitazone. Curr Clin Pharmacol. 2017;12(1):41-49. |
|  |  | In Vitro |  | Nicotine and letrozole | Noteworthy interactions | Espiritu MJ et al; Mechanisms of Herb-Drug Interactions Involving Cinnamon and CYP2A6: Focus on Time-Dependent Inhibition by Cinnamaldehyde and 2-Methoxycinnamaldehyde. Drug Metab Dispos. 2020 Oct;48(10):1028-1043. |
| 30 | *Terminalia arjuna* (ROXB.) WIGHT & ARN. (Arjun) | In Vitro | Arjuna | Metoprolol succinate | Significant reduction in AUC0-24h and Cmax | Varghese A et al; In Vitro CYP2D Inhibitory Effect and Influence on Pharmacokinetics and Pharmacodynamic Parameters of Metoprolol Succinate by Terminalia arjuna in Rats. Drug Metab Lett. 2016;10(2):124-35. |
|  |  | In Vitro, In Vivo |  | Phenacetin | No significant change | Varghese A et al; In vitro and in vivo Evaluation of CYP1A Interaction Potential of Terminalia Arjuna Bark. Indian J Pharm Sci. 2014 Mar;76(2):138-47. |
| 31 | *Ocimum sanctum* L. (Basil) | In Vivo | Surasa | Levetiracetam | Exerted better seizure control, memory retention, oxidative stress reduction, and neuronal structure preservation | Sarangi SC et al; An interaction study of Ocimum sanctum L. and levetiracetam in pentylenetetrazole kindling model of epilepsy. J Ethnopharmacol. 2020 Mar 1;249:112389. |
|  |  | In Vivo |  | Valproate | Better neurobehavioral function and reduced oxidative stress | Sarangi SC et al; Pharmacokinetic and pharmacodynamic interaction of hydroalcoholic extract of Ocimum sanctum with valproate. Epilepsy Behav. 2017 Oct;75:203-209. |
| 32 | *Centella asiatica* (L.) URBAN (Gotu kola) | In Vivo | Mandukaparni | Phenytoin, valproate and gabapentin | Additive effect | Vattanajun A et al; Isobolographically additive anticonvulsant activity between Centella asiatica's ethyl acetate fraction and some antiepileptic drugs. J Med Assoc Thai. 2005 Nov;88 Suppl 3:S131-40. |
|  |  | In Vivo |  | Valproate and phenytoin | Improve the therapeutic efficacy | Kumar R et al;  Pharmacodynamic and pharmacokinetic interactions of hydroalcoholic leaf extract of Centella asiatica with valproate and phenytoin in experimental models of epilepsy in rats. J Ethnopharmacol. 2021 Apr 24;270:113784. |
|  |  | Review |  | Amitriptyline | Enhanced bioavailability of amitriptyline, hypothesized to be mediated by the inhibition of CYP3A4 and CYP2D6 in rat models | Bernardo J, Valentão P. Herb-drug interactions: A short review on central and peripheral nervous system drugs. Phytother Res. 2024 Apr;38(4):1903-1931. doi: 10.1002/ptr.8120. Epub 2024 Feb 15. PMID: 38358734. |
| 33 | *Phyllanthus amarus* SCHUM. & THENN. (Niruri) | In Vivo | Bhūmyāmalaki | Midazolam | Increased oral bioavailability of MDZ | Taesotikul T et al; Effects of Phyllanthus amarus on the pharmacokinetics of midazolam and cytochrome P450 activities in rats. Xenobiotica. 2012 Jul;42(7):641-8. |
|  |  | In Vitro |  | 5-Fluorouracil | Change the levels of RNs (rna) and dRNs (drna) | Guo JR et al; Effect of Phyllanthus amarus Extract on 5-Fluorouracil-Induced Perturbations in Ribonucleotide and Deoxyribonucleotide Pools in HepG2 Cell Line. Molecules. 2016 Sep 20;21(9):1254. |
| 34 | *Allium cepa* L. (Onion) | Review | Palāṇḍu | Warfarin, hepatotoxically acting medicaments, MAOI inhibitors, phenelzin sulphate, or phenytoin | Decrease or completely eliminate the therapeutic effect of the administered drugs | Tůmová L. Interakce mezi lécivými rostlinami a lécivy [Interactions between herbal medicines and drugs]. Ceska Slov Farm. 2000 Jul;49(4):162-7. |
|  |  | Review |  | Warfarin | Increase the risk of bleeding or potentiate the effects of warfarin therapy | Heck AM et al; Potential interactions between alternative therapies and warfarin. Am J Health Syst Pharm. 2000 Jul 1;57(13):1221-7; |
| 35 | *Azadirachta indica* A. JUSS. (Neem) | Review | Nimba | Gliclazide, glipizide |  | Thikekar AK et al; Herb-drug interactions in diabetes mellitus: A review based on pre-clinical and clinical data. Phytother Res. 2021 Sep;35(9):4763-4781. |
|  |  | In Vivo |  | Glipizide | Decrease the bioavailability | Chaudhari S et al; Effect of Aqueous Extract of Azadirachta indica Leaves on Pharmacokineics and Pharmacodynamics of Glipizide. Drug Metab Lett. 2019;13(1):19-24. |
| 36 | *Tinospora cordifolia* (WILLD.) HOOK.F. & THOMS. (Indian Giloy) | Review | Guḍūci | Glibenclamide, glimepiride |  | Thikekar AK et al; Herb-drug interactions in diabetes mellitus: A review based on pre-clinical and clinical data. Phytother Res. 2021 Sep;35(9):4763-4781. |
|  |  | In Vivo |  | Glibenclamide | Increase in the bio availability with a significant delay of Tmax and suppression of clearance. | Sahu R et al; Effect of Tinospora cordifolia aqua-alcoholic extract on pharmacokinetic of Glibenclamide in rat: An herb-drug interaction study. J Pharm Biomed Anal. 2018 Mar 20;151:310-316. |
| 37 | *Boswellia serrata* ROXB. (Indian olibanum tree) | Review | Śallaki | Warfarin |  | Milić N et al; Warfarin interactions with medicinal herbs. Nat Prod Commun. 2014 Aug;9(8):1211-6. |
|  |  | Review |  | Glimepiride |  | Thikekar AK et al; Herb-drug interactions in diabetes mellitus: A review based on pre-clinical and clinical data. Phytother Res. 2021 Sep;35(9):4763-4781. |
| 38 | *Terminalia bellerica* ROXB. (Beleric myrobalan) | Review | Vibhitaki | Diltiazem | CYP3A4 CYP2D6 | Shaikh AS et al; Herb-drug interaction studies of herbs used in treatment of cardiovascular disorders-A narrative review of preclinical and clinical studies. Phytother Res. 2020 May;34(5):1008-1026. |
|  |  | In Vitro |  | Novobiocin | Significantly enhanced the activity | Phatthalung PN et al; Thai ethnomedicinal plants as resistant modifying agents for combating Acinetobacter baumannii infections. BMC Complement Altern Med. 2012 Apr 26;12:56. |
| 39 | *Tamarindus indica* L. (Tamarind) | Review | Ciñca | Aspirin | Enhances the bleeding risk | Abebe W. Herbal medication: potential for adverse interactions with analgesic drugs. J Clin Pharm Ther. 2002 Dec;27(6):391-401. |
|  |  | In Vitro |  | Tamoxifen | Complete elimination of the cytotoxic inhibition effect of tamoxifen and the plant extract | Guneidy RA et al; Antioxidant or pro-oxidant and glutathione transferase P1-1 inhibiting activities for Tamarindus indica seeds and their cytotoxic effect on MCF-7 cancer cell line. J Genet Eng Biotechnol. 2020 Nov 19;18(1):74. |
| 40 | *Cassia senna* L. (Senna) | Review | Svarnapatri | ………… | Decreases drug absorption | Fugh-Berman A. Herb-drug interactions. Lancet. 2000 Jan 8;355(9198):134-8. |
| 41 | *Cassia senna* L and *C.angustifolia* (Senna) |  |  | Thiazide diuretics and corticosteroids | Recommendation- Exercise caution | Williamson EM. Interactions between herbal and conventional medicines. Expert Opin Drug Saf. 2005 Mar;4(2):355-78. |
|  |  | Case Report |  | Digoxin | Increased the digoxin toxicity | Beltrá-Picó I, Díaz-González M, Nalda-Molina R, Ramon-Lopez A, Pascual-Bartolomé S, Miralles-Macià CF, Rodríguez-Soler M, Más-Serrano P. Cassia angustifolia and tacrolimus interaction in a liver transplant patient, a case report. Br J Clin Pharmacol. 2024 Jul;90(7):1745-1750. doi: 10.1111/bcp.16079. Epub 2024 Apr 24. PMID: 38657592 |
| 42 | *Terminalia chebula* RETZ. & WILLD. (Chebulic myrobalan) | In Vitro | Haritaki | Novobiocin | Significantly enhanced the activity | Phatthalung PN et al; Thai ethnomedicinal plants as resistant modifying agents for combating Acinetobacter baumannii infections. BMC Complement Altern Med. 2012 Apr 26;12:56. |
| 43 | *Bacopa monnieri* (L.) PENNELL (Water hyssop) | Review | Brahmi | Neurological and Psychotic drugs |  | Wilson V et al; Herb-Drug Interactions in Neurological Disorders: A Critical Appraisal. Curr Drug Metab. 2018;19(5):443-453. |
|  |  | Review |  | Amitriptyline | The pre-treatment with *B. monnieri* enhanced the oral bioavailability of amitriptyline as verified by a significant increase in C_max_, AUC and t_1/2_ | Bernardo J, Valentão P. Herb-drug interactions: A short review on central and peripheral nervous system drugs. Phytother Res. 2024 Apr;38(4):1903-1931. doi: 10.1002/ptr.8120. Epub 2024 Feb 15. PMID: 38358734. |
| 44 | *Linum usitatissimum* L. (Flaxseed) | Review | Atasī | Laxatives | Flaxseed may cause diarrhea and should be taken with [58] ample water to prevent constipation and intestinal obstruction | Vahabi S et al; Phyto-anesthetics: A mini-review on herb-anesthesia drug interactions. Biomed Pharmacother. 2016 Dec;84:1885-1890. |
| 45 | *Citrus aurantium* L.( Sour Orange) | In Vivo |  | Amiodarone | Increase of the peak plasma concentration | Investigating herb-drug interactions: the effect of Citrus aurantium fruit extract on the pharmacokinetics of amiodarone in rats. Food Chem Toxicol. 2013 Oct;60:153-9. |
| 46 | *Mangifera indica* L. (Anacardiaceae) (Mango) and *Carica papaya* L. (Caricaceae) | Review | Āmra and eraṇḍa karkaṭi | Warfarin | Anticoagulant effect increased | Skalli S et al; Drug interactions with herbal medicines. Ther Drug Monit. 2007 Dec;29(6):679-86. |
| 47 | *Swertia chirata* (ROXB. EX. FLEM.) KAR. (Chiretta) | Review | Kirātatikta | Tolbutamide |  | Thikekar AK et al; Herb-drug interactions in diabetes mellitus: A review based on pre-clinical and clinical data. Phytother Res. 2021 Sep;35(9):4763-4781. |
| 48 | *Commiphora myrrha* ENGL. (Myrrh) | In Vivo | Guggulu | Theophylline | Alters the pharmacokinetics of theophylline. | Al-Jenoobi FI et al; Investigating the Potential Effect of Commiphora myrrha on the Pharmacokinetics of Theophylline, a Narrow Therapeutic Index Drug. Drug Res (Stuttg). 2015 Jun;65(6):312-6. |
| 49 | *Garcinia cambogia* GAERTN. DESR. (Malabar Tamarind) | In Vivo | Vṛkṣāmla | Lamotrigine | Significant decrease in the rate of systemic exposure; increase of apparent volume of distribution | Ventura S et al; Short-term effects of Garcinia cambogia extract on the pharmacokinetics of lamotrigine given as a single-dose in Wistar rats. Food Chem Toxicol. 2019 Jun;128:61-67. |
| 50 | *Phyllanthus embilica* L. (Indian gooseberry) | Clinical Trial | Āmalaki | Clopidogrel and ecosprin | Significant antiplatelet activity | Fatima N et al; Study of pharmacodynamic interaction of Phyllanthus emblica extract with clopidogrel and ecosprin in patients with type II diabetes mellitus. Phytomedicine. 2014 Apr 15;21(5):579-85. |
| 51 | *Piper betel* L. (Betel leaf) | In Vitro | Tāmbūla patra | 5-fluorouracil | Enhances the cytotoxicity effect in inhibiting the growth of HT29 and HCT116 colon cancer cells | Ng PL et al; Piper betle leaf extract enhances the cytotoxicity effect of 5-fluorouracil in inhibiting the growth of HT29 and HCT116 colon cancer cells. J Zhejiang Univ Sci B. 2014 Aug;15(8):692-700. |
| 52 | *Hibiscus rosa-sinesis* L. (Hibiscus) | In Vitro | Japa | Taxol, cisplatin, and tamoxifen | Enhance the induction of apoptosis of chemotherapy; increase oxidative stress and decrease mitochondrial membrane potential | Nguyen C et al; Hibiscus flower extract selectively induces apoptosis in breast cancer cells and positively interacts with common chemotherapeutics. BMC Complement Altern Med. 2019 May 6;19(1):98. |
| 53 | *Artemisia absinthium* L. (Common Wormwood) | Case Report | Damanaka | Warfarin | Elevated INR | Açıkgöz SK et al; Gastrointestinal bleeding secondary to interaction of Artemisia absinthium with warfarin. Drug Metabol Drug Interact. 2013;28(3):187-9. |
| 54 | *Psoralea corylifolia* L. (Babchi) | In Vitro | Bākuci | Paracetamol/acetaminophen | Significantly increased hepatotoxicity | Britza SM et al; Paracetamol (acetaminophen) hepatotoxicity increases in the presence of an added herbal compound. Leg Med (Tokyo). 2020 Nov;47:101740. |
|  |  | Review |  | Propofol | Interactions may strongly reduce its clearance, increase its half-life, and extend its anaesthetic or toxic effects. | Bernardo J, Valentão P. Herb-drug interactions: A short review on central and peripheral nervous system drugs. Phytother Res. 2024 Apr;38(4):1903-1931. doi: 10.1002/ptr.8120. Epub 2024 Feb 15. PMID: 38358734. |
| 55 | *Foeniculum vulgare G*AERTN. (Sweet fennel) | In Vivo | Śatapuṣpa | Ciprofloxacin | Absorption, distribution and elimination of ciprofloxacin were all affected | Zhu M et al; Effect of oral administration of fennel (Foeniculum vulgare) on ciprofloxacin absorption and disposition in the rat. J Pharm Pharmacol. 1999 Dec;51(12):1391-6. |
|  |  |  |  | Acetaminophen | Inhibits the oxidation | Langhammer AJ et al; Fennel and raspberry leaf as possible inhibitors of acetaminophen oxidation. Phytother Res. 2014 Oct;28(10):1573-6. |
| 56 | *Pimpinella anisum* L. |  |  | Warfarin | Increase the risk of bleeding or potentiate the effects of warfarin therapy | Heck AM et al; Potential interactions between alternative therapies and warfarin. Am J Health Syst Pharm. 2000 Jul 1;57(13):1221-7; |
| 57 | *Ferula assafoetida L.* (Asafoetida) | Review | Hiṅgu | Warfarin | Increase the risk of bleeding or potentiate the effects of warfarin therapy | Heck AM et al; Potential interactions between alternative therapies and warfarin. Am J Health Syst Pharm. 2000 Jul 1;57(13):1221-7; |
| 58 | *Syzygium aromaticum* (L.) MERRILL & PERRY (Clove) | Review | Lavaṅga | Warfarin | Increase the risk of bleeding or potentiate the effects of warfarin therapy | Heck AM et al; Potential interactions between alternative therapies and warfarin. Am J Health Syst Pharm. 2000 Jul 1;57(13):1221-7; |
|  |  | In Vivo |  | Ketorolac | Synergized the antinociceptive effect | Beltrán-Villalobos KL et al; Synergistic antinociceptive interaction of Syzygium aromaticum or Rosmarinus officinalis coadministered with ketorolac in rats. Biomed Pharmacother. 2017 Oct;94:858-864. |
|  |  | Review |  | Propofol | Enhances propofol by acting as allosteric activators of benzodiazepine binding sites on the GABA_A_ receptor | Bernardo J, Valentão P. Herb-drug interactions: A short review on central and peripheral nervous system drugs. Phytother Res. 2024 Apr;38(4):1903-1931. doi: 10.1002/ptr.8120. Epub 2024 Feb 15. PMID: 38358734. |
| 59 | *Eucalyptus globulus* LABILL. (Blue gum) | In Vivo | Tailaparni | Benzodiazepine | Had an inhibitory effect at both doses and could be useful at the highest dose in cases where the desired effect of the depressant is moderate anxiolytic activity without marked muscle relaxation | Quílez AM et al; Uncaria tomentosa (Willd. ex. Roem. & Schult.) DC. and Eucalyptus globulus Labill. interactions when administered with diazepam. Phytother Res. 2012 Mar;26(3):458-61. |
| 60 | *Ziziphus jujuba* LM. (Jujube) | In Vivo | Badara | Phenacetin | Decreased concentrations of the drugs | Jing XY et al; Effects of Ziziphus jujuba fruit extracts on cytochrome P450 (CYP1A2) activity in rats. Chin J Nat Med. 2015 Aug;13(8):588-94. |
|  |  | Review |  | Anticholinergic | Increase anticholinergic burden. | Bernardo J, Valentão P. Herb-drug interactions: A short review on central and peripheral nervous system drugs. Phytother Res. 2024 Apr;38(4):1903-1931. doi: 10.1002/ptr.8120. Epub 2024 Feb 15. PMID: 38358734. |
| 61 | *Beta vulgaris* L. (Beetroot) | In Vitro | Raktagṛnjana | Doxorubicin | Overall positive reduction in drug concentration was achieved by D when combined with B in its cytotoxicity profile | Kapadia GJ et al; Synergistic cytotoxicity of red beetroot (Beta vulgaris L.) extract with doxorubicin in human pancreatic, breast and prostate cancer cell lines. J Complement Integr Med. 2013 Jun 26;10: |
| 62 | *Holarrhena pubescens* (BUCH.-HAM.) WALLICH EX DO (Kurchi) | In Vitro | Kutaja | Novobiocin | Significantly enhanced the activity | Phatthalung PN et al; Thai ethnomedicinal plants as resistant modifying agents for combating Acinetobacter baumannii infections. BMC Complement Altern Med. 2012 Apr 26;12:56. |
| 63 | *Gloriosa superba* L. (Flame lily) | In Vivo | Lāṅgali | Gemcitabine | Added value | Capistrano I R et al; Coadministration of a Gloriosa superba extract improves the in vivo antitumoural activity of gemcitabine in a murine pancreatic tumour model. Phytomedicine. 2016 Nov 15;23(12):1434-1440. |
| 64 | *Cassia auriculata L.* (Mature Tea Tree) | In Vivo | Avartaki | Metformin | Dose level of MT may be reduced to produce the same therapeutic effect | Elango H et al; Assessment of Pharmacodynamic and Pharmacokinetic Interaction of Aqueous Extract of Cassia auriculata L. and Metformin in Rats. Pharmacogn Mag. 2015 Oct;11(Suppl 3):S423-6. |
| 65 | *Alpinia officinarum* HANCE. (Lesser galangal) | In Vitro | Rāsna | Ceftazidime | Synergism | Eumkeb G et al; Reversing β-lactam antibiotic resistance of Staphylococcus aureus with galangin from Alpinia officinarum Hance and synergism with ceftazidime. Phytomedicine. 2010 Dec 15;18(1):40-5. |
| 66 | *Psidium guajava* L. var. pomiferum L. (Guava) | In Vivo | Peruka | Warfarin | Enhanced the anticoagulant activity | Alnaqeeb M et al; Critical pharmacokinetic and pharmacodynamic drug-herb interactions in rats between warfarin and pomegranate peel or guava leaves extracts. BMC Complement Altern Med. 2019 Jan 24;19(1):29. |
| 67 | *Taraxacum officinale* WIGGERS.(Milk-Witch) | In Vivo | Payasvini | Dasatinib, imatinib and nilotinib |  | Alzoman NZ et al; UPLC-MS/MS study of the effect of dandelion root extract on the plasma levels of the selected irreversible tyrosine kinase inhibitors dasatinib, imatinib and nilotinib in rats: Potential risk of pharmacokinetic interactions. Biomed Chromatogr. 2019 Dec;33(12):e4674. |
| 68 | *Ocimum basilicum* L. (Basil) | In Vitro | Surasa | Rifampicin | Metabolism of the drug | Kumar S et al; In Vitro Assessment of the Interaction Potential of Ocimum basilicum (L.) Extracts on CYP2B6, 3A4, and Rifampicin Metabolism. Front Pharmacol. 2020 Apr 30;11:517. |
| 69 | *Brassica oleracea* L. (Cabbage) | In Vivo | Kembuka | Midazolam, caffeine, and dextromethorphan | Significant increase in plasma levels and the AUC | Yamasaki I et al; Inhibitory effects of kale ingestion on metabolism by cytochrome P450 enzymes in rats. Biomed Res. 2012;33(4):235-42. |
| 70 | *Solanum nigrum* L. (Black Nightshade) | Review | Kākamāci | Doxorubicin | Potentiates the drug through autophagy | Lin SR et al; Natural compounds as potential adjuvants to cancer therapy: Preclinical evidence. Br J Pharmacol. 2020 Mar;177(6):1409-1423. |
| 71 | *Raphanus sativus* L. (Radish) | Review | Mūlaka | Anticholinergic drugs | Increase anticholinergic burden. | Bernardo J, Valentão P. Herb-drug interactions: A short review on central and peripheral nervous system drugs. Phytother Res. 2024 Apr;38(4):1903-1931. doi: 10.1002/ptr.8120. Epub 2024 Feb 15. PMID: 38358734. |
| 72 | *Citrus reticulata* BLANCO  (Manderin orange) | Review | Naranga | Anticholinergic drugs | Increase anticholinergic burden. | Bernardo J, Valentão P. Herb-drug interactions: A short review on central and peripheral nervous system drugs. Phytother Res. 2024 Apr;38(4):1903-1931. doi: 10.1002/ptr.8120. Epub 2024 Feb 15. PMID: 38358734. |
| 73 | *Myristica fragrans* Houtt. (Nutmeg) | Review | Jatipatri | Propofol | Enhances propofol by acting as allosteric activators of benzodiazepine binding sites on the GABA_A_ receptor | Bernardo J, Valentão P. Herb-drug interactions: A short review on central and peripheral nervous system drugs. Phytother Res. 2024 Apr;38(4):1903-1931. doi: 10.1002/ptr.8120. Epub 2024 Feb 15. PMID: 38358734. |
| 74 | *Cinnamomum verum* PRESL (Cinnamon) | Review | Tamālapatra | Propofol | Enhances propofol by acting as allosteric activators of benzodiazepine binding sites on the GABA_A_ receptor | Bernardo J, Valentão P. Herb-drug interactions: A short review on central and peripheral nervous system drugs. Phytother Res. 2024 Apr;38(4):1903-1931. doi: 10.1002/ptr.8120. Epub 2024 Feb 15. PMID: 38358734. |
| 75 | *Plantago ovata* Forssk (Isaphgul seeds) | Review | Aśvakarṇa | Lithium | Significantly reduce the bioavailability of lithium, which can be restored if its consume is discontinued, or at least, patients wait several hours before taking lithium. | Bernardo J, Valentão P. Herb-drug interactions: A short review on central and peripheral nervous system drugs. Phytother Res. 2024 Apr;38(4):1903-1931. doi: 10.1002/ptr.8120. Epub 2024 Feb 15. PMID: 38358734. |
| 76 | *Syzygium cumini* (L.) Skeels (Java plum) | Review | Jambu | Buspirone | The intestinal transport of buspirone was reduced via CYP3A4 inhibition | Bernardo J, Valentão P. Herb-drug interactions: A short review on central and peripheral nervous system drugs. Phytother Res. 2024 Apr;38(4):1903-1931. doi: 10.1002/ptr.8120. Epub 2024 Feb 15. PMID: 38358734. |
| 77 | *Murraya koenigii* (L.) Spreng. (Curry leaf) | Review | Kālaśāka | Buspirone | The intestinal transport of buspirone was reduced via CYP3A4 inhibition | Bernardo J, Valentão P. Herb-drug interactions: A short review on central and peripheral nervous system drugs. Phytother Res. 2024 Apr;38(4):1903-1931. doi: 10.1002/ptr.8120. Epub 2024 Feb 15. PMID: 38358734. |
